# Population heterogeneity in clinical cohorts affects the predictive accuracy of brain imaging

**DOI:** 10.1371/journal.pbio.3001627

**Published:** 2022-04-29

**Authors:** Oualid Benkarim, Casey Paquola, Bo-yong Park, Valeria Kebets, Seok-Jun Hong, Reinder Vos de Wael, Shaoshi Zhang, B. T. Thomas Yeo, Michael Eickenberg, Tian Ge, Jean-Baptiste Poline, Boris C. Bernhardt, Danilo Bzdok

**Affiliations:** 1 McConnell Brain Imaging Centre (BIC), Montreal Neurological Institute (MNI), Faculty of Medicine, McGill University, Montreal, Canada; 2 Institute of Neuroscience and Medicine (INM-1), Forschungszentrum Jülich, Jülich, Germany; 3 Department of Data Science, Inha University, Incheon, South Korea; 4 Center for Neuroscience Imaging Research, Institute for Basic Science, Sungkyunkwan University, Suwon, South Korea; 5 Center for the Developing Brain, Child Mind Institute, New York, New York, United States of America; 6 Department of Biomedical Engineering, Sungkyunkwan University, Suwon, South Korea; 7 Department of Electrical and Computer Engineering, National University of Singapore, Singapore, Singapore; 8 Centre for Sleep and Cognition (CSC) & Centre for Translational Magnetic Resonance Research (TMR), National University of Singapore, Singapore, Singapore; 9 N.1 Institute for Health & Institute for Digital Medicine (WisDM), National University of Singapore, Singapore, Singapore; 10 Flatiron Institute, New York, New York, United States of America; 11 Psychiatric and Neurodevelopmental Genetics Unit, Center for Genomic Medicine, Massachusetts General Hospital, Boston, Massachusetts, United States of America; 12 Department of Biomedical Engineering, Faculty of Medicine, McGill University, Montreal, Canada; 13 School of Computer Science, McGill University, Montreal, Canada; 14 Mila—Quebec Artificial Intelligence Institute, Montreal, Canada; University of Cambridge, UNITED KINGDOM

## Abstract

Brain imaging research enjoys increasing adoption of supervised machine learning for single-participant disease classification. Yet, the success of these algorithms likely depends on population diversity, including demographic differences and other factors that may be outside of primary scientific interest. Here, we capitalize on propensity scores as a composite confound index to quantify diversity due to major sources of population variation. We delineate the impact of population heterogeneity on the predictive accuracy and pattern stability in 2 separate clinical cohorts: the Autism Brain Imaging Data Exchange (ABIDE, *n* = 297) and the Healthy Brain Network (HBN, *n* = 551). Across various analysis scenarios, our results uncover the extent to which cross-validated prediction performances are interlocked with diversity. The instability of extracted brain patterns attributable to diversity is located preferentially in regions part of the default mode network. Collectively, our findings highlight the limitations of prevailing deconfounding practices in mitigating the full consequences of population diversity.

## Abstract 2

Brain-imaging research enjoys increasing adoption of supervised machine learning for single-subject disease classification. This study explores the contribution of diversity-aware machine learning models to tracking, unpacking and understanding out-of-distribution generalization in large-scale neuroimaging datasets, and shows that population diversity is a key factor contributing to generalization performance.

## Introduction

Brain scanning technology opens a noninvasive window into the structure and function of the human brain. Combined with machine learning algorithms, brain imaging research is now gaining momentum to transition from group-level contrast analyses toward single-participant prediction [1–3]. In supervised learning, the main purpose is to learn coherent patterns from brain measurements (i.e., brain signatures) that can be used to make accurate forecasts for new participants [4,5]. The prediction paradigm holds the promise of improving disease diagnosis, enhancing prognostic estimates, and ultimately paving the way to precision medicine [6,7]. Machine learning methods are now increasingly adopted for the goal of classifying various conditions, including autism spectrum disorder (ASD) [8–11], attention-deficit/hyperactivity disorder (ADHD) [12–15], anxiety (ANX) [16,17], or schizophrenia [18–22]. However, there is a large variability in the diagnostic accuracy reported across studies [23–25]. Counterintuitively, the larger the clinical cohorts have become, the worse the prediction performance of machine learning algorithms has become. The unknown reasons behind this varying success hamper the translation of emerging neuroimaging findings into clinical practice.

In the current landscape of machine learning applications in neuroimaging, researchers are facing challenges related to the generalizability and replicability of brain patterns valuable for prediction. Careful validation of machine learning tools is therefore needed to ascertain the robustness of the learned brain patterns. When using small and homogeneous datasets, however, common cross-validation (CV) procedures are more likely to yield high prediction accuracies, although at the expense of producing biased biomarkers with poor generalization to new or future cohorts. The last 2 decades have witnessed an unprecedented growth in the number of open-access data-sharing initiatives in the neuroscientific community [26–31]. In these initiatives, data are collected from multiple acquisition sites to aggregate larger and demographically more heterogeneous datasets. Such multisite data pooling efforts are more likely to reflect the true diversity in the wider populations. This first wave of retrospective data collection initiatives offers great opportunities for the advancement of scientific discoveries in neuroscience [32,33].

However, these efforts also pose new hurdles due to the diverging characteristics and backgrounds of the participants who are recruited at each site. Discrepancies in demographic and clinical variables may interfere with the identification of robust relationships between imaging-based features and the target variables (e.g., diagnosis). These challenges may entail potential bias for downstream decision-making by healthcare professionals and other stakeholders. Moreover, gathering brain scans from multiple sites comes with the potential cost of increased heterogeneity due to different sources of variation, such as batch effects [34,35]. Although the higher heterogeneity in multisite, compared to single site, cohorts may provide better generalization to participants from unseen scanning sites, classification in multisite cohorts has generally shown lower performance [23,36–38]. When handled with insufficient care, these general sources of variation that tell people apart may prevent predictive models from learning accurate patterns and weaken their performance. For these reasons, faithful disease classification in multisite cohorts may increasingly require sharp tools that take several dimensions of diversity into account.

Previous studies have typically focused on assessing generalizability along a single dimension, for example, evaluating a trained predictive model on a specific subset of participants from unseen scanning sites (e.g., leave-N-sites-out), a subset of participants with a specific sex or age range (e.g., [39–42]). The increasingly embraced CV tactics can monitor and probe the effects from one specific source of heterogeneity. Yet, cohort heterogeneity may stem from multiple aspects of diversity (e.g., age, sex, and acquisition site). For example, a performance decay in held-out acquisition sites might be in part attributable to other aspects of population variation (e.g., age and sex distribution). To deal with such diversity, we need approaches that can appropriately incorporate sources of population heterogeneity that should be acknowledged in the analytical workflow (cf [43]).

Our study aims to provide investigators with a handle to directly quantify the ramifications of population diversity. We propose to recast the propensity score framework to faithfully track out-of-distribution behavior of predictive analysis pipelines. Briefly, the propensity score has originally been introduced for computing the probability of treatment assignment given an individual’s collective covariates [44]. An appealing property of propensity scores is the opportunity to encapsulate a mixed envelope of covariates and express them as a single dimension of variation. Once built, a propensity score model can be deployed for a variety of purposes, including matching, stratification, covariate adjustment, and weighting [45,46]. Henceforth, our notion of diversity relies on continuous differences in the propensity scores of the participants in a cohort. Participants who are closer to each other on the propensity score spectrum can be assumed to have more similar constellations of covariates. Larger differences in propensity scores indicate bigger gaps in population stratification or background between a given pair of participants. After stratifying the cohort by means of propensity scores, we examined different sampling schemes to analyze the impact of diversity on the performance of our predictive models and assess their generalizability. It is worth noting here that the purpose of our study was not to improve the generalization abilities of these predictive models [7,47], but rather to provide a principled way to characterize population diversity. Hence, our aim was to investigate and understand the role of diversity in brain imaging prediction (i.e., is there an impact of common dimensions of population variation on neuroimaging-based machine learning?). For the estimation of propensity scores and thus diversity, we considered a set of nonimaging covariates with potential confounding effects that are widely used and readily available in most neuroimaging datasets [48–50]: age, sex assigned at birth, and scanning site. Results on the classification of participants with a neurodevelopmental diagnosis versus healthy controls from both the Autism Brain Imaging Data Exchange (ABIDE) and Healthy Brain Network (HBN) cohorts using functional and structural brain measurements show that, even after rigorous matching and nuisance deconfounding, diversity exerts a substantial impact on the generalization accuracy of the predictive models and on the pattern stability of the extracted biomarkers.

## Methods

### Participants and brain scanning resources

We examined resting-state functional MRI (rs-fMRI) and cortical thickness data from both waves of the openly shared ABIDE initiative (ABIDE I and II; http://fcon_1000.projects.nitrc.org/indi/abide) [29,30] and the HBN dataset [26], releases 1–8 collected between June 2017 and January 2020.

For ABIDE, we considered data from all acquisition sites with at least 10 participants per group and with both children and adults. After detailed quality control, only cases with acceptable T1-weighted (T1w) MRI, surface extraction, and head motion in rs-fMRI were included. Participants with ASD were diagnosed based on an in-person interview administered by board-certified mental health professionals using the gold standard diagnostics of the Autism Diagnostic Observation Schedule (ADOS) [51] and/or Autism Diagnostic Interview-Revised (ADI-R) [52,53]. Typically developing (TD) participants had no history of mental disorders. For all groups, participants were excluded on the basis of genetic disorders associated with autism (i.e., Fragile X), contraindications to MRI scanning, or current pregnancy. The ABIDE data collections were performed in accordance with local Institutional Review Board guidelines, and data were fully anonymized. These quality procedures resulted in a total of 297 participants (151 ASD and 146 TD) from 4 different sites (i.e., New York University Langone Medical Center [NYU], University of Pittsburgh, School of Medicine [PITT], University of Utah, School of Medicine [USM], and Trinity Centre for Health Sciences [TCD]) in ABIDE.

For HBN, we included 102 TD individuals and 449 participants carrying a diagnosis of ASD, ADHD, and/or ANX. From all participants with at least one diagnosis, 53 were diagnosed with ASD and ADHD, 12 diagnosed with ASD and ANX, 99 with ANX and ADHD, and 17 participants were diagnosed with all 3. Only 24 ASD, 171 ADHD, and 73 ANX participants had no comorbidity. The HBN cohort offers a broad range of heterogeneity in developmental psychopathology. Participants were recruited using a community referral model (for inclusion criteria, see https://fcon_1000.projects.nitrc.org/indi/cmi_healthy_brain_network/Recruitement.html#inclusion-exclusion). Psychiatric diagnoses were assessed and reported by clinicians according to DSM-5 criteria [54]. HBN was approved by the Chesapeake Institutional Review Board. Written informed consent was obtained from all participants and from legal guardians of participants younger than 18 years. Hence, after assessing the same criteria for quality control, we ended up with a total of 551 participants, with several participants having more than one neurodevelopmental diagnosis, from 3 different sites (i.e., Staten Island [SI], Rutgers University Brain Imaging Center [RU], and CitiGroup Corcell Brain Imaging Center [CBIC]) in HBN.

More details about the demographic information of the participants included in our study and about acquisition settings used for both datasets are reported in **Table 1** and **S1 Table**, respectively.

**Table 1:** Demographics for each site and group.

	Site	*N*				Sex (M/F)				Age			
		TD	ASD	ADHD	ANX	TD	ASD	ADHD	ANX	TD	ASD	ADHD	ANX
**ABIDE**	**NYU**	70	56	-	-	69/1	52/4	-	-	15.4 ± 7.0	14.5 ± 7.9	-	-
	**PITT**	22	20	-	-	22/-	20/-	-	-	19.7 ± 7.0	20.8 ± 7.3	-	-
	**TCD**	19	18	-	-	19/-	18/-	-	-	15.8 ± 3.2	14.5 ± 3.3	-	-
	**USM**	40	52	-	-	40/-	52/-	-	-	21.5 ± 7.8	23.6 ± 7.6	-	-
	**All**	151	146	-	-	150/1	142/4	-	-	17.7 ± 7.3	18.6 ± 8.4	-	-
**HBN**	**CBIC**	23	41	172	86	11/12	37/4	119/53	52/34	11.7 ± 3.3	12.5 ± 4.0	11.5 ± 3.3	12.4 ± 3.7
	**RU**	33	49	130	74	19/14	36/13	87/43	43/31	11.6 ± 3.5	12.7 ± 4.0	11.6 ± 3.3	12.1 ± 3.5
	**SI**	46	16	38	41	20/26	11/5	24/14	23/18	11.8 ± 3.7	12.6 ± 3.4	12.8 ± 4.1	12.8 ± 3.8
	**All**	102	106	340	201	50/52	84/22	230/110	118/83	11.7 ± 3.5	12.6 ± 3.9	11.7 ± 3.4	12.4 ± 3.6

Number of participants (*N*), males/females (M/F), and mean age and standard deviation for each site and group. Note that some participants from HBN are diagnosed with more than one disorder.

ABIDE, Autism Brain Imaging Data Exchange; ADHD, attention-deficit/hyperactivity disorder; ANX, anxiety; ASD, autism spectrum disorder; CBIC, CitiGroup Corcell Brain Imaging Center; HBN, Healthy Brain Network; NYU, New York University Langone Medical Center; PITT, University of Pittsburgh, School of Medicine; RU, Rutgers University Brain Imaging Center; SI, Staten Island; TCD, Trinity Centre for Health Sciences, Trinity College Dublin; TD, typically developing; USM, University of Utah, School of Medicine.

### Brain scan preprocessing workflow

For both ABIDE and HBN datasets, structural brain scans (T1w MRI) were preprocessed with FreeSurfer [55–57]. The pipeline performed automated bias field correction, registration to stereotaxic space, intensity normalization, skull stripping, and tissue segmentation. White and pial surfaces were reconstructed using triangular surface tessellation and topology corrected. Surfaces were inflated and spherically registered to the fsaverage5 template. Segmentations and surfaces were subject to visual inspection. Participants with erroneous segmentations were excluded from further analysis.

For the resting-state functional brain scans in ABIDE, we built on data previously made available by the Preprocessed Connectomes initiative (http://preprocessed-connectomes-project.org/abide). The preprocessing was performed with C-PAC (https://fcp-indi.github.io) and included slice time correction, head motion correction, skull stripping, and intensity normalization. The rs-fMRI data were detrended and adjusted for common nuisance effects related to head motion, white matter, and cerebrospinal fluid signals using CompCor [58], followed by band-pass filtering (0.01 to 0.1 Hz). For the rs-fMRI data in HBN, we discarded the first 5 volumes, removed the skull, and corrected for head motion. Magnetic field inhomogeneity was corrected using topup with reversed phase-encoded data [59]. After applying a high-pass filter at 0.01 Hz, nuisance effects were removed using ICA-FIX [60]. We excluded participants who had mean framewise displacement greater than 0.3. Individual rs-fMRI data were mapped to the corresponding mid-thickness surfaces, resampled to the Conte69 template (https://github.com/Washington-University/Pipelines), and smoothed using a 5 mm full-width-at-half-maximum (FWHM) kernel.

### Pattern learning pipeline for disease classification

The construction of the feature space from the brain scans was based on a topographical brain parcellation: 100 functionally defined cortical regions from a widely used reference atlas [61]. For cortical thickness, each participant’s fingerprint consisted of a 100-feature vector corresponding to the mean cortical thickness of the vertices within each of the 100 atlas regions. Similarly, for intrinsic functional fluctuations, the time series were averaged over all vertices in each of the 100 atlas regions and used to build functional connectivity profiles based on all pairwise correlations (Pearson correlation coefficient) between the 100 region time series. For the rs-fMRI, the feature vectors thus consisted of 4,950 unique region–region connectivity strengths.

The predictive model we used for classification (e.g., ASD versus TD) is the commonly used logistic regression model with an optimization loss that includes a penalty term for Tikhonov (*l*_2_) regularization. Let *w* be the coefficients (i.e., weights) of our model and *x* the feature vector (e.g., of cortical thickness values) corresponding to a given participant. For prediction of continuous class assignments in form of log odds, the model specification involves mapping the weighted combination of features (i.e., *z* = *w*^*T*^*x*) from input space (cf feature engineering above) into a probability using the sigmoid function. For gradient descent–based estimation of this convex optimization problem, the set of final model coefficients indicating the global minimum was obtained by solving a structured risk minimization problem based on the following cost function:

$$J(w)=-\sum_{i}^{n}[y^{\left(i\right)}log(\varphi (z^{\left(i\right)}))+(1-y^{\left(i\right)})log(1-\varphi (z^{\left(i\right)}))]+\lambda \|w{\|}^{2},$$

where *n* is the number of observations, *y*^(*i*)^ carries the class label (i.e., ASD or TD), *z*^(*i*)^ is the weighted combination of features of the *i*-th observation, and *φ* denotes the sigmoid function, which is defined as *φ*(*z*) = 1/(1+*e*^−*z*^). The first term is the log-likelihood function, and the second term penalizes the weight coefficients, with *λ* denoting the hyperparameter that controls the regularization strength of the *l*_2_ penalty term. Tikhonov regularization is known to exert smooth parameter shrinkage by acting on the low-variation directions that underlie the input space relatively more than the dominant high-variation patterns in the data [62]. This model has been shown to perform better than several competing machine learning models in comprehensive benchmark studies of ASD classification [39] and other commonly studied phenotype predictions [41,63].

To rigorously evaluate our analytical workflow, we adopted a nested CV strategy. Regardless of how the outer CV was implemented (see paragraph below on out-of-distribution prediction), we consistently used an inner CV based on 5-fold for tuning the hyperparameter controlling the regularization strength (i.e., *λ*). The value for the complexity parameter *λ* was chosen from a grid of 7 equidistant points in logarithmic scale in the interval [1e-3, 1e+3]. Prior to fitting our model to the data, brain features were z-scored across participants on the training set at hand (i.e., respecting the mechanics of CV), and the derived parameters were then used to apply z-scoring of the features to the participants in the test set.

### Quantifying diversity using propensity scores

Our overarching aim was to characterize the role of population heterogeneity in forming prediction by means of pattern extraction. The notion of diversity used throughout this work leans on 3 important facets of population variation that are available in most population datasets: participant age, participant sex, and scanning site. In our study, we proposed to get a handle on this notion of participant diversity by virtue of the propensity score framework that is established in a variety of domains [44]. Diversity, thus, denoted the distance based on these common covariates between participants in our cohorts. Observational studies have benefited from propensity scores to successfully estimate the probability of a participant receiving treatment given the covariates. Here, we concentrated on estimating the probability that a given participant carries a disease diagnosis based on a set of covariates, i.e.,

π=P(Y=1|C),

where *Y* is the target variable, with *Y* = 1 for ASD and *Y* = 0 for TD. *C* are the covariates, which collectively provided the basis for quantifying population diversity. In other words, we estimated a model for the purpose of deriving a propensity score as a function of the covariates of a given participant. Although other machine learning tools have sometimes been used to obtain propensity scores [64–66], logistic regression is probably the most common implementation in this context. This natural choice of method yields the following model specification for our propensity scores framework:

$$logit(\pi )=\beta_{0}+\beta_{1}C_{1}+\beta_{2}C_{2}+\cdots +\beta_{k}C_{k},$$

where *k* denotes the number of covariates or sources of population variation, *β*_0_ is the intercept capturing the average classification log odds in the participant data at hand, while *logit*(·) is a nonlinear transformation (called “link function” in the generalized linear modeling family) that maps from probability space to log odds space. In other words, we invoked a conditional expectation that is implemented by a logistic regression model to predict diagnosis (e.g., ASD) using the covariates of each participant as features. To prevent class imbalance from skewing the probabilities, we further used probability calibration based on Platt scaling [67] to produce the final propensity scores. Although propensity scores are often based on treatment assignment, for simplicity, we use them throughout this work to refer to the probability we defined above. Note that the propensity scores were estimated using a conventional logistic regression model based solely on the participant covariates as input variables—without any access to brain imaging information. In contrast, the predictive model used for the actual disease classification problem (see Pattern learning pipeline for disease classification section) employed a regularized logistic regression with the l2-norm to predict disease diagnosis based on brain imaging features (e.g., functional connectivity). We reiterate that the propensity score model itself did not involve or had access to any of the brain imaging features of primary scientific interest.

A key asset of the propensity score framework is the opportunity to seamlessly construct subsets of participants with homogeneous background profiles with respect to the observed covariates. This follows because the propensity score is a balancing score. At each value *p* of the propensity score, the distributions of the covariates are the same in both groups [44,68], i.e.,

P(C|π(C)=p,Y=1)=P(C|π(C)=p,Y=0),

where *π*(*C*) denotes the propensity score corresponding to the covariate set *C*, and Y is a binary variable holding the diagnosis.

Multiple applications of propensity scores can be used for confounding adjustment including matching, stratification and inverse probability weighting [45,46]. In previous neuroimaging studies, propensity scores have been used to address confounding in permutation testing [69], classification [70], and regression [49] problems. Note that, unlike these methods, our proposed approach leverages propensity scores to investigate the relationship between population variation and out-of-distribution prediction rather than to perform deconfounding. An appealing quality of propensity scores is a form of dimensionality reduction: representing a mixed envelope of covariates as a single number that coherently encapsulates the sources of interparticipant variation indexed by the joint covariates. Matching, for example, can then be performed directly on the propensity scores rather than on the covariates, which rapidly becomes infeasible with high-dimensional vectors of covariates. Moreover, propensity scores can be readily used to handle both continuous (e.g., age) and categorical (e.g., site or sex) covariates in a single coherent framework. Any possible collinearity in coefficient estimation of the propensity score model does not affect the results because this modeling step follows the prediction goal [47]. In other words, we are not concerned with point estimate, interval estimate, or subject matter interpretation of any specific coefficient inside the propensity score model itself [71].

After estimating the propensity scores, participants were spanned out along a one-dimensional continuum that gauges the diversity among them: The more dissimilar the participants in their covariates, the more distant they were from each other on the propensity score spectrum. By chunking the participants into strata with similar confounding architecture, within each of which participants had minimal diversity relative to each other, we could assess the generalizability of our predictive models sampling different strata for train and test in an operationalizable series of experiments.

### Matching and stratification

Naively partitioning the data points, however, may produce strata with unbalanced classes. To safeguard against skewing of results due to class imbalance, we initially matched participants based on their propensity score estimates. We employed a pair matching approach: One specific participant in the TD group was allocated to one participant in the ASD group. To identify these pairs in a data-driven fashion, we carried out the matching procedure using the Hungarian algorithm [72]. The Hungarian algorithm obtained a globally optimal pairing by minimizing the global distance (i.e., a “cost” quantity) between paired participants based on the absolute difference of their estimated propensity scores. Nonetheless, this approach is not exhaustive, in that, the absolute differences between matched participants are unlikely to be zero. This circumstance especially comes into play when there is imperfect overlap between the overall distributions of estimated propensity scores in ASD and TD. This is because there is a risk of ending up with paired participants with very dissimilar propensity scores and hence different positioning according to directions of population variation.

**Fig 1:**
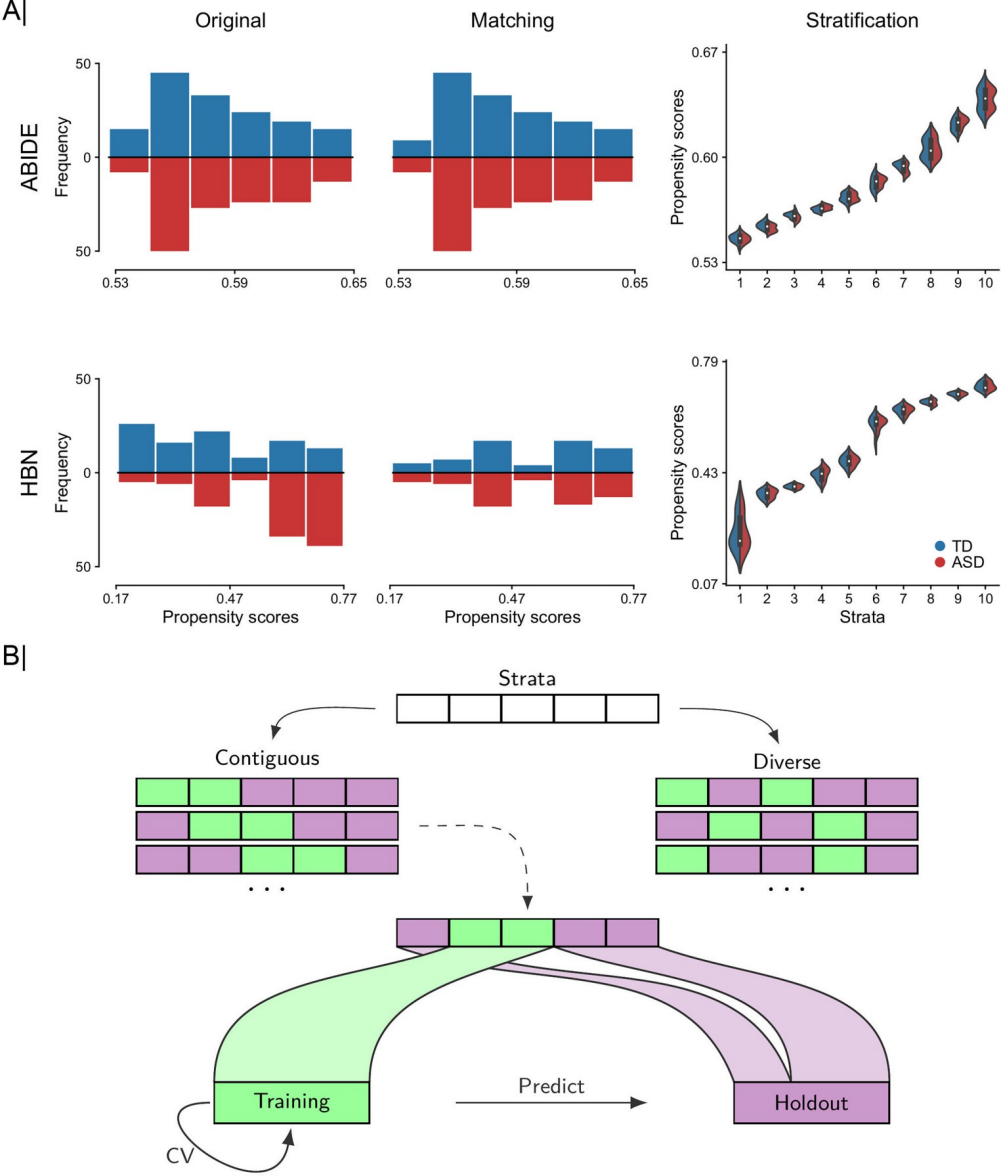
Workflow for systematic participant stratification and diversity-based participant sampling. We repurpose propensity scores as a tool to translate several different indicators of population variation into a single diversity index. **(A)** From left to right: 2-sided histograms in which we show bin counts bins of estimated propensity score distributions for TD (blue) and ASD (red) participants before (i.e., using all observations in original dataset) and after matching (based on age, sex, and site) and partitioning of matched participants in 10 equally sized participant sets (i.e., strata) based on similarities of their propensity scores for ABIDE (top) and HBN (bottom) cohorts. Distributions of propensity scores after matching showed better overlap (middle panel) than the unmatched original distributions (left panel), especially in HBN. Propensity scores are computed as a function of age, sex, and site, which are commonly available in many future population cohorts. **(B)** Diversity-based participant sampling: Given the attribution of each participant to 1 of *q* homogeneous strata, a subset of *r* strata is picked and combined to form the training set used for estimating the predictive model (green) and the *q*-*r* remaining strata served as held-out set for testing the performance of that learning model (violet). Two different sampling regimes are used to form the training set: (i) a contiguous scheme where the training set is composed of adjacent strata of similar diversity; and (ii) a diverse scheme where the training data are composed of noncontiguous (at least one) strata with participants pulled from diverse populations. For each analysis setting, the classification accuracy is assessed within the training set based on rigorous 10-fold fit–tune–predict CV cycles, and then all the training data are used to predict disease status in unseen holdout participants. Data underlying this figure can be found in S1 Data. ABIDE, Autism Brain Imaging Data Exchange; ASD, autism spectrum disorder; CV, cross-validation; HBN, Healthy Brain Network; TD, typically developing.

To overcome this hurdle, we added a restriction to the Hungarian algorithm such that the absolute difference in the propensity scores of matched participants must not exceed a prespecified threshold—the so-called caliper [73]. This constraint is commonly achieved by setting the cost of matching pairs of participants to a large value when their difference exceeds the threshold. Then, paired participants returned by the Hungarian algorithm are inspected to discard those pairs that exceed the threshold. The value must be larger than all values in the cost matrix of the Hungarian algorithm. In this work, the caliper was set to 0.2, which is a popular choice in the literature [74,75]. The maximum acceptable distance for pairs of participants to be considered a match was then set to 0.2*d_*sd*_, where *d*_*sd*_ was the standard deviation of all pairwise absolute differences between the participants in our dataset. The benefit of using a caliper was that we achieved a better matching of the considered participants, at the possible expense of reducing the number of participants in a data analysis scenario. Thus, final pairs of TD–ASD participants had very similar covariates. As shown in **Fig 1A**, distributions of propensity scores after matching showed better overlap (middle panel) than the unmatched original distributions (left panel), especially in HBN. The demographic information of the participants after matching is reported in **S2 Table**. The quality of matching was assessed using standardized mean difference (SMD). SMD is a widely used diagnostic to examine the balance in the covariates between the groups (i.e., ASD and TD). For a continuous covariate, the absolute SMD is defined as

$$d=\frac{\left|x_{1}-x_{0}\right|}{\sqrt{{{(s}_{1}}^{2}+{s_{0}}^{2})/2}},$$

where *x*_*i*_ and $s_{i}^{2}$ denote the mean and variance of the covariate in the group indexed by subscript *i* (*i* = 0 for TD, and *i* = 1 for ASD), respectively. For binary variables,

$$d=\frac{\left|p_{1}-p_{0}\right|}{\sqrt{(p_{1}(1-p_{1})+p_{0}(1-p_{0}))/2}},$$

where *p*_*i*_ denotes the prevalence in each group. Usually, a covariate with an SMD <0.1 is considered well balanced [46]. As shown in **S1 Fig**, matching on the propensity scores allowed us to achieve balance in both ABIDE and HBN. The only exception was the sex covariate in ABIDE, although its SMD was very close from 0.1. Moreover, for our analyses, this was unlikely to have any impact because the results because the proportion of females in ABIDE was negligible. After matching, we ended up with 290 participants (145 participants per group) in ABIDE and 126 (63 participants per group) in HBN, which were used subsequently for the classification of ASD versus TD in both datasets.

Based on the matched data, the aligned participant pairs were ranked according to their mean estimated propensity scores (i.e., the mean propensity score of a matched pair of participants) and stratified into mutually exclusive subsets, henceforth “strata.” In our work, participants were divided into 10 equally sized strata using the deciles of the estimated propensity scores. Although matching based on the mean propensity score of paired participants may not be perfect in all cases, **Fig 1A** shows good overlap between the distributions of propensity scores for each group (i.e., ASD and TD) within each of the 10 strata. In ABIDE, in all participants, we obtained propensity scores above 0.5. Propensity scores above 0.5 show that participants had demographic properties, captured by the covariate configurations alone, which were more indicative of the disease group. Nonetheless, using high propensity scores was unlikely to have any impact on our disease classification results because we are not interested in the values of the propensity scores per se, but rather in their distance. In other words, diversity was the same when participants had the same distance between their propensity scores, regardless of their positions on the propensity score spectrum. Moreover, as shown in **S1 Fig**, we achieved strong balance with matching, indicating that the estimated propensity scores provided a robust and useful proxy for the underlying covariates. In HBN, the nonuniform distribution of sex across the strata, with females only at the lowest end of the propensity score spectrum, corroborated the validity of our approach to characterize diversity. Strata containing female participants were closer to each other (lower diversity) than to strata containing only male participants (higher diversity). Note that, in the present work, the reason behind stratifying the participants in our dataset based on their estimated propensity scores was different from that used in observational studies, where stratification can be employed for example to estimate stratum-specific effects. Stratification was here repurposed as a tool for the sole aim of training and testing predictive models on sets of participants whose similarities and differences we could control in our data analysis experiments.

### Out-of-distribution prediction

To illustrate the role of diversity in single-participant prediction, we started by comparing distinct participant sampling schemes when forming the training and holdout sets. In the contiguous scheme, we only selected adjacent strata for training and the remaining strata were “kept in a vault” for later testing of the generalizability in classifying ASD from TD. In the diverse scheme, the training set was composed of (at least one) noncontiguous strata. As a baseline, we furthermore used a random scheme where training data were drawn by taking into account the class proportions but irrespective of their propensity scores or strata. The random scheme amounted to a conventional CV strategy.

This analysis was repeated for different sizes of the training set, ranging from 2 to 8 strata. In the random scheme, we hence pulled 20% to 80% of the total participants in our dataset for training of the predictive model. For each number of strata (or subset of participants in case of random sampling) used for training, we performed 20 draws and computed the average of the ensuing model performances. In the diverse scheme, we did not consider all possible combinations of training/test strata, but rather focused on the combinations that provided the most diverse strata for training. For each diverse draw (i.e., combination of training strata with at least one nonadjacent stratum), we computed the mean absolute difference in propensity scores of the participants in the training strata (i.e., within-distribution diversity). We ranked the draws according to their within-distribution diversity and then chose the top 20 draws. In the contiguous scheme, the number of possible draws was less than 20 (e.g., 9 maximum draws when using 2 contiguous strata for training).

To ensure a consistent number of draws, additional draws with similar size for the training set (e.g., 20% of pairs when using 2 strata for training) were sampled by considering paired participants in consecutive order (according to their propensity scores) for training and the remaining for holdout. These sampling schemes allowed us to compare out-of-distribution (i.e., participants with unseen propensity scores) performance when we learned from data that spanned the wider space parameterized by propensity scores (i.e., diverse), focused on a narrow subspace (i.e., contiguous), or completely ignored this kind of information in predictive modeling (i.e., random). Moreover, we also assessed performance in the within-distribution setting. That is, based solely on the training strata, we used a 10-fold CV to evaluate performance when our model was trained and tested using participants with similar propensity scores. Although train and test participants had similar propensity scores when assessing within-distribution performance, predictive performance was not based on the same participant data used to build the predictive models.

Note that the matching step was important to prevent the contiguous and diverse sampling schemes from producing unbalanced train/test splits that may potentially bias our results. Without matching, we would have ended up with substantially unbalanced strata, especially in the HBN dataset (see **Fig 1A**). In the case of imbalance in the training strata, the predictive models would have likely overpredicted toward the majority class. On the other hand, an imbalance in the testing strata would have favored predictive models that tend toward the majority class and also required the consideration of additional evaluation metrics (e.g., specificity, sensitivity, precision, etc.). More importantly, there might have been draws with imbalance in both train and test data, although inverted when going from training to the testing stage. This would have occurred, for example, in the unmatched HBN dataset when using the strata with the lowest/highest propensity scores for training/testing. Here, the proportion of TD would have been substantially higher/lower in the training/testing strata. In this case, we would not have been able to tell if the observed prediction accuracy was related to the diversity in the data, to the imbalance in the strata, or even to both. With matching, we avoided class imbalance and ensured that changes in prediction accuracy were due to diversity.

### Assessing the relationship of performance with diversity

The previous analyses offered an initial depiction of the implications of participant diversity on out-of-distribution performance. Yet, the analysis only considered 20 draws of training/test strata. In such cases, the contiguous and diverse sampling schemes may have been dominated by the strata at both extremes of the diversity spectrum, i.e., those strata of pulled participants with the lowest/highest propensity scores. These strata were likely to be part of the test set in most draws in the contiguous scheme (because only adjacent strata were used for training). In the diverse scheme, on the other hand, these strata were likely to be part of the training set (because they were the most distant strata). The overrepresentation of the strata at both ends of the propensity score spectrum may have contributed to the obtained constellation of findings in terms of performance as well as in terms of the stability of the predictive patterns. Furthermore, diversity was experimentally controlled with a focus on the training set, rather than the holdout participants. In another set of analyses, we examined the relationship between model performance and diversity for each draw of training/test strata. To this end, we used 5 strata for training (and the remaining 5 for holdout) and considered all possible combinations of participant strata to form the training set (i.e., 252 different draws, picking 5 out of 10 strata to form the training set).

Within-distribution performance was reported based on the average performance using a 10-fold CV strategy, whereas out-of-distribution performance was reported separately for each holdout stratum. For assessment of within-distribution performance, diversity was computed as the average of all pairwise absolute differences in propensity scores:

$$\frac{2}{N_{t}(N_{t}-1)}\sum_{i,j,i>j}^{N_{t}}\left|\pi_{i}-\pi_{j}\right|,$$

where *N*_*t*_ is the total number of participants in the training strata, and *π*_*i*_ denotes the propensity score of the *i*-th participant. For the purpose of out-of-distribution prediction, diversity denotes the mean absolute difference in propensity scores between all pairs of participants in the training set and those in the holdout stratum:

$$\frac{1}{N_{t}N_{s}}\sum_{i}^{N_{t}}\sum_{j}^{N_{s}}\left|\pi_{i}-\pi_{j}\right|,$$

where *N*_*s*_ is the number of participants in the holdout stratum. This analysis allowed us to provide a clearer look into the impact of diversity on predictive modeling, by directly inspecting the relationship between participant diversity and predictive accuracy. Note that we assessed out-of-distribution performance for each stratum separately to prevent that sampling variation due to the diversity in the testing data (using all testing strata) influences our CV procedure. Stratification assigned individuals with similar covariates to the same stratum. Therefore, when evaluating performance of a built predictive model in a single holdout stratum, the remaining diversity among the participants in the stratum (as captured by covariate configurations) was substantially minimized and unlikely to impact performance. Moreover, diversity was roughly the same across all strata. On the other hand, if we had used the participants in all the testing strata to report performance, our results would be potentially biased because the diversity in the testing data substantially changed from one draw to another (as the strata used for testing change). Therefore, reporting out-of-distribution performance for each single holdout stratum ensured that differences in prediction accuracy were not participant to the diversity among testing participants, but only to the diversity between the training and testing participants.

### Relation to common deconfounding practices

The covariates used in this study to investigate the impact of diversity on the classification of ASD may have a confounding effect in how the brain scanning measurements are used to predict diagnostic categories. In fact, numerous earlier studies have tried to account for nuisance sources by deconfounding the imaging-derived variables in what is often thought as a data cleaning step [43,76–78]. For example, considering the sex of the participants, we could find that males are more prone to be classified as ASD than females. This can be seen, for example, in the distribution of sex across the different strata in the HBN dataset (see **S2 Fig**). We noted that females occurred in the strata with the lowest propensity scores, while only males were in the highest propensity score strata. Regarding scanning site as a dimension of diversity, the variance of the brain imaging–derived variables may differ from one site to another. This could lead to a biased estimation of the underlying ASD-related pattern in the brain since the model may have learned differences in variance rather than the true putative pattern [48,79]. In such scenarios, it is important to seek to remove these sources of confounding.

To shed light on these circumstances, we explored (i) a conventional linear regression model; and (ii) ComBat [80] for the purpose of variable deconfounding before the actual predictive model of interest. The first method is a standard approach to control for confounds in neuroimaging. It estimates the effect of confounds using a linear regression model to then remove their contribution to the data. The residualized functional connectivity strengths or regional morphological measurements then served as input variables of interest fed into the quantitative model of interest, such as a machine learning model [78,81]. For categorical confounds such as the imaging site, this amounts to centering the data within each site, without considering the potential intersite differences in variance [48]. To deal with the latter point, we also carried out ComBat: a recently proposed approach for better correcting intersite effects [80,82]. ComBat has been previously applied to multiple neuroimaging data modalities including cortical thickness [82,83], resting-state functional connectivity [35,84], and diffusion tensor imaging [82,85]. Briefly, ComBat involves Bayesian hierarchical regression modeling to estimate and remove effects attributable to acquisition site additive and structured noise from the brain imaging features. ComBat might also remove the effects of other factors (e.g., age and sex) if they are colinear with site. For example, if age covaried to a certain extent with site, ComBat may remove some variation related to age when removing site effects [43]. However, we cannot rely on ComBat to remove the variation related to other factors. Here, in addition to the site covariate used for harmonization, our ComBat model further incorporated the age and sex covariates. Thus, the ComBat-based deconfounding used in our work was composed of 2 steps: (1) remove site-specific effects while preserving variation related to age and sex using ComBat, and then (2) regress out age and sex effects using conventional linear regression.

We performed deconfounding using all participants in our dataset, prior to matching and running our analysis. Note that in alternative settings, to prevent data leakage, only the training data should be used to build the deconfounding model, without considering the test data [86]. However, the main reason behind our choice was that deconfounding in data points matched based on the same covariates may have little impact on the results [70], as shown in **S3 Fig**.

### Dissecting the impact of cohort diversity

To provide a richer picture into how diversity affects disease classification, we next used confusion matrices to tease apart the cases in which our predictive models succeeded or failed. Moreover, prediction success may be contingent on the different covariates used to build the propensity scores. Here, we laid out the implications of diversity by taking into consideration the covariates of the holdout participants. Given a particular holdout stratum, prediction performance was assessed for participants from each of the different scanning sites. The analogous procedure was carried out to study sex. We also inspected the changes in performance that were attributable to age differences between the participants in the training set and each holdout stratum.

Finally, we analyzed the degree to which diversity affects the stability of the extracted predictive patterns. To this end, we performed the analysis at both the regional (i.e., 100 target regions) and network levels (i.e., 7 target canonical networks) as defined by the widely used Schaefer–Yeo atlas [61,87]. First, we extracted the coefficients (i.e., weight vectors) from our 252 predictive model instances (corresponding to all possible combinations of picking 5 out of 10 strata to form the training set). These model coefficients were then assigned to 5 different groups according to the diversity of the training set used to learn the models (i.e., the average of all pairwise absolute differences in propensity scores of the participants in the training set). We tested for significant coefficient differences between groups using classical 1-way analysis of variance (ANOVA) with 5 levels (i.e., 5 groups: from very low to very high diversity, no continuous input variables). At the region level, we ended up with 252 coefficients corresponding to our collection of models. Differences in coefficient estimates were analyzed on a region-by-region basis using separate ANOVAs. This approach allowed identifying the cortical regions whose variance in predictive contributions (across the 252 previously obtained predictive models) could be partitioned according to the diversity factor levels in a statistically defensible fashion. Note that in the case of functional connectivity, we used model coefficients for each node (average of the regression coefficients across all edges of a node), and the threshold-free cluster enhancement approach was used with 1,000 permutations to correct for multiple comparisons [88]. At the network level, we analyzed the relationships between model coefficients and diversity by computing Pearson correlation coefficient between the network-aggregated coefficients of predictive models and the diversity of the training participants (i.e., the average of all the pairwise absolute differences in propensity scores of the training observations). These systematic assessments were aimed to uncover which parts of the brain are most at stake when predictive models from heterogeneous participant observations are to be interpreted by the neuroscientific investigator.

## Results

In this work, we explored the value of propensity scores as a handle to detect and monitor the role of cohort diversity in predictive modeling. With our approach, we were able to encompass multiple sources of population variation into a single dimension that meticulously recapitulated the diversity among the participants in our cohorts. Tailoring the propensity score framework to brain imaging predictions allowed us to bring to the fore the relationship between diversity and prediction accuracy in a rigorous manner. More specifically, we investigated how diversity plays out in classification of neuropsychiatric disorders using structural and functional brain imaging profiles constructed from the ABIDE [29,30] and the HBN [26] datasets. Specific site inclusion criteria and data quality control resulted in a total of 297 participants (151/146 ASD/TD) from 4 different acquisition sites in ABIDE and 208 participants (106/102 ASD/TD) from 3 sites in HBN (**S1 and S2 Tables**). Our image processing strategy involved the mapping of functional signals to cortical surfaces as well as surface-based alignment. Functional connectivity matrices were calculated at a single-participant level based on a widely used parcellation atlas with 100 cortical regions [61].

Prior to predictive modeling, ASD and TD participants were matched and grouped into strata with homogeneous backgrounds based on their propensity scores (see **Fig 1**). This step yielded a total of 290 participants (145 participants per group) in ABIDE and 126 in HBN. In ABIDE, a very small number of participants was discarded from subsequent analyses after matching by age, sex, and acquisition site. However, with HBN around one-third of the original participants was excluded because of the modest overlap between the distributions of estimated propensity scores between TD and ASD groups. In **Fig 1A**, we can see that in HBN the distribution of TD participants was skewed to the right, while for ASD it was skewed to the left. In HBN, we further explored the role of diversity in the participant classification of ADHD versus TD and ANX versus TD. After matching, we submitted a total of 180 (90 ADHD/TD) and 188 (94 ANX/TD) participants to each classification analysis, respectively. Further information about the datasets, image processing, matching, and classification settings is provided in the Methods section.

### Benchmarking out-of-distribution prediction performance

**Fig 2:**
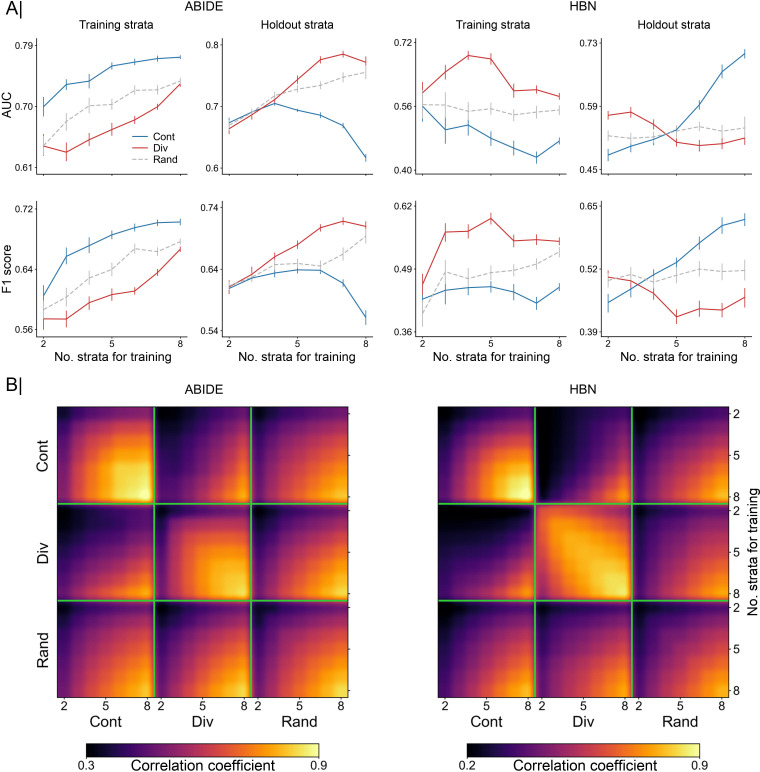
Accuracy of out-of-distribution prediction and consistency of extracted predictive patterns. Results are based on the classification of ASD versus TD using functional connectivity profiles. **(A)** Comparison of model accuracy based on contiguous (Cont) and diverse (Div) training sets in the classification of autism. Prediction performance is reported as AUC (top) and F1 score (bottom) in 2 separate cohorts: ABIDE (left) and HBN (right). Accuracy is assessed using different training sets sized from 2 to 8 combined strata. For each cohort, the first column indicates the prediction accuracy using a 10-fold CV strategy based solely on participants from the training strata. Folds were randomly sampled, without considering the propensity scores of the participants. The second column displays the performance in the holdout strata, which contains the remaining participants (from untouched strata). As a baseline for comparison, we used an additional sampling scheme (Rand): Participants for the training set were randomly chosen regardless of their propensity scores. **(B)** Consistency of model coefficients was quantified by Pearson correlation coefficient across 2 given models in the contiguous, diverse, and random data scenarios. For each of these sampling schemes, consistency is shown for different numbers of combined strata used for predictive model training (from 2 to 8 combined strata), delineated by the green segments. Building models based on diverse strata of participants entailed considerable differences in predictive patterns from those learned based on models with similar participants (i.e., contiguous). Data underlying this figure can be found in S1 Data. ABIDE, Autism Brain Imaging Data Exchange; ASD, autism spectrum disorder; AUC, area under the curve; CV, cross-validation; HBN, Healthy Brain Network; TD, typically developing.

Results in the classification of ASD versus TD participants in both ABIDE and HBN datasets are shown in **Fig 2**, with performance reported in terms of area under the receiver operating characteristic curve (hereafter, AUC) and F1 score. In the ABIDE cohort, when we trained and tested on data with similar distributions (first column), the contiguous scheme reached better prediction performance than the diverse scheme, with the random scheme lying in between. This trend remained stable as we increased the number of strata used for training. However, we can observe in the holdout dataset (second column) that the difference in prediction accuracy showed an inverted trend. The predictive model trained on the contiguous strata showed poor generalization abilities. We note a clear decay in classification performance as we increased the number of strata used for training, which may suggest that the model was overfitting to participants that share similar demographic status (e.g., age and sex), and hence may have been limited to generalize to more diverse data. On the other hand, the diverse and random schemes performed better as we grew the size of the training set. In the HBN dataset, we observed the opposite trends with the diverse analysis scenario, which showed higher performance within-distribution and poor generalizability.

In the context of these diverging results obtained for ABIDE and HBN, we note the difference in the occurrence of females in each dataset. In HBN, 36% of participants were females (45/126), whereas in ABIDE, the proportion of females was negligible (<0.02%, 5/290). Sex showed an expected strong contribution to the computation of diversity (through the propensity scores) and to its relationship with prediction accuracy in HBN. In ABIDE, however, the sex covariate had almost no contribution (see **S4 Fig**). Thus, the difference in the sex covariate between HBN and ABIDE (with practically no females in ABIDE) may be a potential factor behind the opposite results in these datasets. Nonetheless, in both datasets, we observed a strong impact of diversity on model performance. This finding indicates that within-distribution performance is not in all cases an accurate proxy for out-of-distribution performance when there is a shift in the distribution of the covariates.

Such tendency received further support from our results in classifying ASD versus TD using cortical thickness in the ABIDE dataset and the other 2 neuropsychiatric disorders (i.e., ADHD versus TD and ANX versus TD) from the HBN dataset based on functional connectivity (see **S5 Fig**). With cortical thickness, we found similar performance trends as the ones obtained with functional connectivity in ABIDE, with the contiguous scheme outperforming the diverse scheme within-distribution but showing lower accuracy in the holdout participants. The same performance pattern was found in ANX. On the other hand, ADHD showed similar trends to the ones found in the classification of ASD in HBN. Model accuracy in the classification of ADHD, however, was very close to 0.5, which may suggest that there may not be enough signal in the data.

We also analyzed the relationship between the functional connectivity patterns (i.e., weights) learned by the models based on the 2 different sampling schemes. More specifically, we calculated the Pearson correlation coefficient to quantify the consistency of model coefficients that we obtained in the scenarios with contiguous, diverse, and random sampling schemes. Results are shown in **Fig 2B** for different sizes of the training set (from 2 to 8 strata for model training) in both ABIDE and HBN datasets for the classification of ASD. With the exception of 2 strata, where the model may not have enough data to extract a robust pattern, the models were able to identify robust patterns (correlation coefficient *r* > 0.7) in both datasets with the diverse sampling. With the contiguous and random schemes, the extracted patterns showed high consistency only when large numbers of strata were used for training, although in ABIDE the contiguous scheme was able to extract stable patterns. This is mainly due to the overlap of the training data, which may relate to our observation of strong pattern stability comparing the sampling schemes when using a large number of strata for training (e.g., 8 strata). Overall, however, the patterns identified by the contiguous and diverse schemes showed very low correlations, which highlights the important role of diversity in obtaining robust and reproducible biomarkers. This was also illustrated by the low pattern correlations of the random scheme, where training participants were chosen without considering the criterion of diversity. **S6 Fig** shows the consistency of patterns obtained in the classification of ASD using cortical thickness in the ABIDE dataset and ADHD and ANX from HBN based on functional connectivity. In accordance with the previous results obtained in the classification of ASD based on functional connectivity, we found a low consistency between the patterns extracted from contiguous and those from diverse strata.

### Charting the tension between prediction accuracy and participant diversity

**Fig 3:**
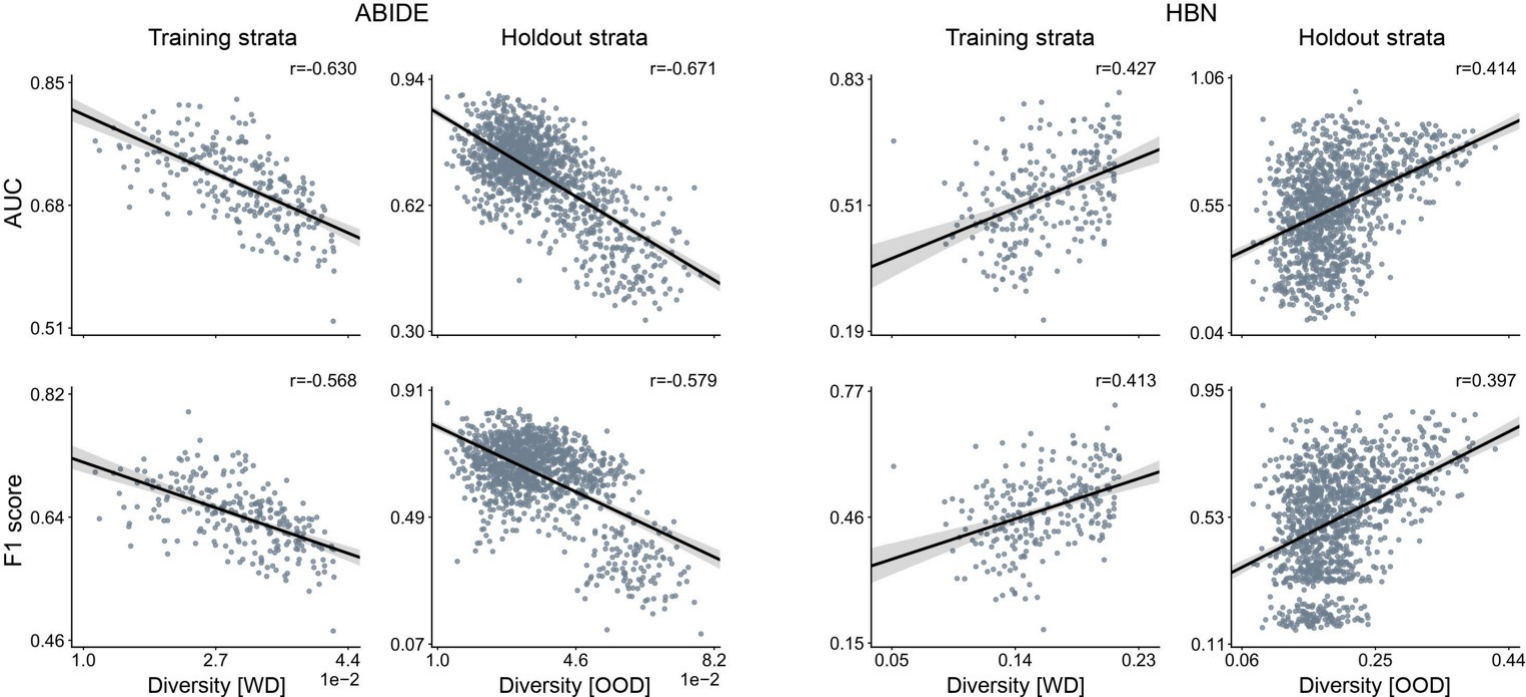
Participant diversity is a major determinant for the classification accuracy of predictive models. Results are based on the classification of ASD versus TD using functional connectivity profiles. For each dataset, results show prediction accuracy for each possible combination of 5 out of 10 strata for training and the remaining 5 strata as holdout. Prediction accuracy based on AUC (top) and F1 score (bottom) in 2 different cohorts: ABIDE (left) and HBN (right). For each cohort, the first column indicates the predictive model performance using a 10-fold CV strategy based solely on the training set, where diversity is computed as the average of all pairwise absolute differences in propensity scores (i.e., WD). The second column displays the performance for each single stratum in the holdout strata. Diversity denotes the mean absolute difference in propensity scores between the participants of the training set and those in the held-out strata with unseen participants (i.e., OOD). The strength of the association between performance and diversity is reported with Pearson correlation coefficient (*r*). Our empirical results show a strong relationship between predictive performance and diversity, although different correlation directions were found in ABIDE and HBN cohorts. Data underlying this figure can be found in S1 Data. ABIDE, Autism Brain Imaging Data Exchange; ASD, autism spectrum disorder; AUC, area under the curve; CV, cross-validation; HBN, Healthy Brain Network; OOD, out of distribution; TD, typically developing; WD, within distribution.

**Fig 3** displays the relationship between model performance and diversity in both ABIDE and HBN datasets in the classification of ASD from functional connectivity. For each cohort, the first column shows within-distribution performance based on a 10-fold CV strategy using only the training set, and the second column shows performance in the holdout data. Results were obtained using 5 strata to build our predictive models (and the remaining 5 for holdout). Performance is reported in terms of AUC and F1 score. With both performance metrics, we can observe a decline in performance in ABIDE as diversity increased in both within (AUC: *r* = −0.630, F1 score: *r* = −0.568) and out-of-distribution (AUC: *r* = −0.671, F1 score: *r* = −0.579) predictions. In the HBN cohort, we found the opposite relationship, with performance being negatively correlated with diversity when assessing both within (AUC: *r* = 0.427, F1 score: *r* = 0.413) and out-of-distribution (AUC: *r* = 0.414, F1 score: *r* = 0.397) predictions. In both datasets, we found strong associations between performance and diversity, although with different signs. In light of the diverging results observed in the 2 cohorts and the negligible number of females in ABIDE, we further inspected the role of diversity in the HBN dataset when considering males and females separately. Diversity was thus only defined by age and scanning site. As illustrated in **S7 Fig**, performance (both within and out-of-distribution) decayed with increasing diversity in both female and male subsets, showing therefore similar trends to that found in the ABIDE cohort. Moreover, **S5 Fig** shows the relationship between performance and diversity in the classification of ASD in ABIDE based on cortical thickness and ADHD and ANX in HBN using functional connectivity. In ADHD, we can observe a positive correlation of performance with diversity. Yet, it was very weak in holdout participants. For the 2 other cases (i.e., ASD versus TD using cortical thickness and ANX versus TD using functional connectivity), we found negative correlations, with performance decreasing the farther the held-out stratum was from the strata used for training.

The aforementioned analysis is based on the stratification of the matched participants, as explained in the Methods section (subsection Matching and stratification). To illustrate the importance of the stratification and matching steps, we further assessed out-of-distribution performance (i.e., holdout strata) without using stratification and without using matching and compared the results to the ones in **Fig 3**. As shown in **S8 and S9 Figs**, respectively, stratification was necessary to produce train–test draws with different propensity scores and allowed us to assess the impact of diversity in performance, while matching was required to prevent class imbalance from biasing results. Furthermore, to better understand the incongruent results obtained from the ABIDE and HBN cohorts when using both males and females, we generated 2 different single-feature toy datasets. In the first synthetic dataset (ABIDE like), the group-wise feature difference decreased as we moved toward the lowest/highest propensity scores, while it increased in the second synthetic dataset (HBN like). Based on these datasets, we assessed the relationship between diversity and AUC using the same approach used in the main analysis. As shown in **S10 Fig**, these results recapitulate the same trends as the ones found in the actual ABIDE and HBN participants and therefore may explain to a great extent the divergent results obtained in ABIDE and HBN.

### Exploring the role of deconfounding practices

**Fig 4:**
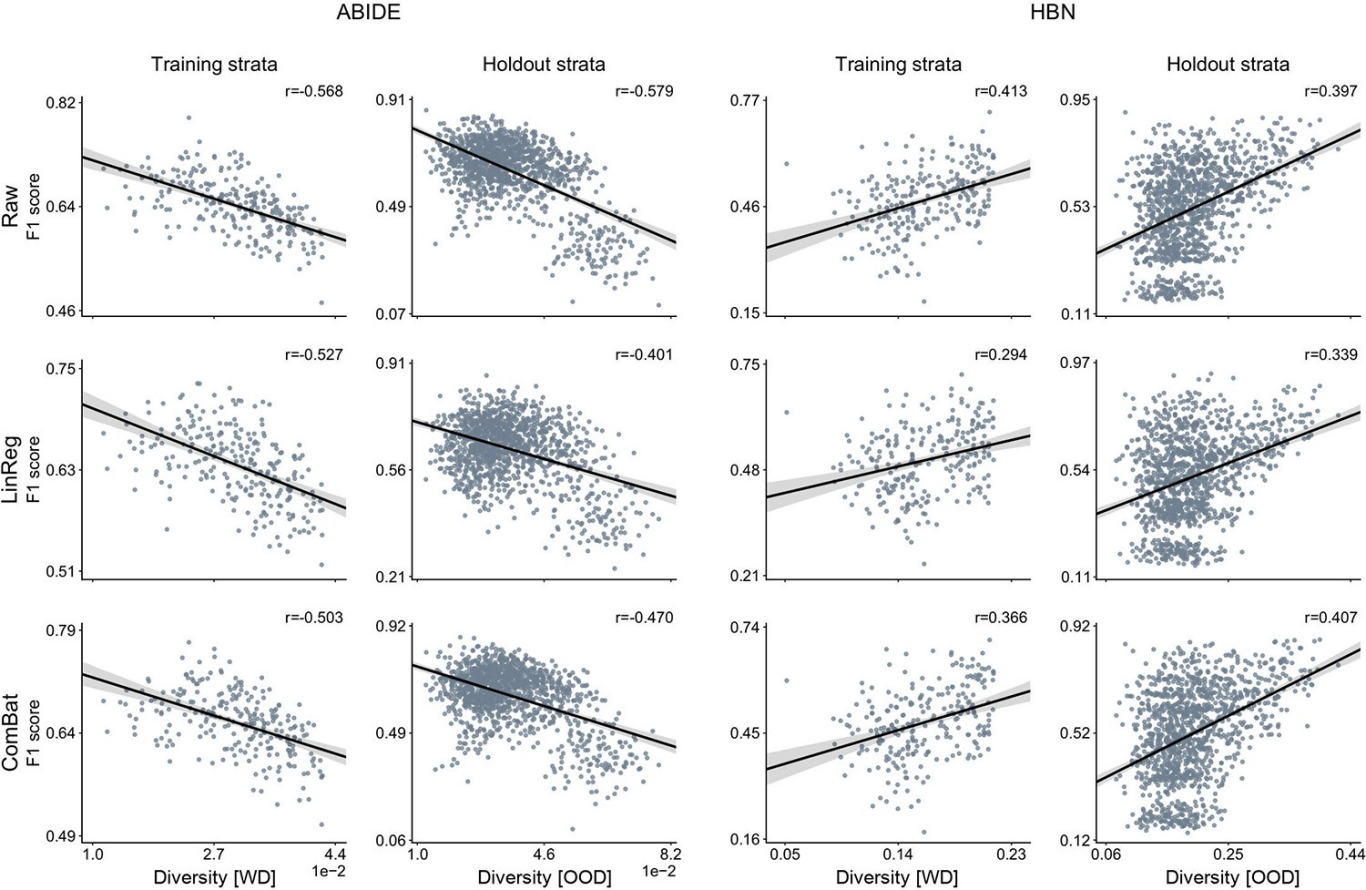
Established and new deconfounding strategies appear insufficient to counteract escalating population diversity. A means to quantify the behavior of predictive models is comparing its predictions on training and testing information. Performance is measured by AUC metric on the original brain imaging data (Raw, top), and after carrying out deconfounding steps on the brain feature prior to the pattern classification pipeline using standard linear regression–based deconfounding (LinReg, middle) and recently proposed ComBat (bottom) approaches. Compared with the conventional nuisance removal using regression residuals, ComBat is a more advanced hierarchical regression to control for site differences. Results are reported for 2 different clinical cohorts: ABIDE (left) and HBN (right). For each cohort, the first column shows the model prediction performance using a 10-fold CV strategy. Diversity is computed as the average of pairwise absolute differences in propensity scores between all participants (i.e., WD). Each dot is a cross-validated accuracy. The second column, for each cohort, displays the performance in the holdout participants. Instead of reporting performance on all the participants in the holdout data, here, performance is assessed independently for participants in each single stratum from the holdout data. Thus, diversity denotes the mean absolute difference in propensity scores between the participants in the training set and those in the held-out stratum (i.e., OOD). Both ComBat and linear regression–based deconfounding failed to mitigate the impact of diversity on prediction accuracy. Data underlying this figure can be found in S1 Data. ABIDE, Autism Brain Imaging Data Exchange; AUC, area under the curve; CV, cross-validation; HBN, Healthy Brain Network; OOD, out of distribution; WD, within distribution.

So far, we have shown that diversity had a considerable impact on model performance, but we did this based on the original raw functional connectivity data. In this section, our aim is to investigate if deconfounding can help us remove, at least to some extent, the impact of diversity on performance. To do so, we used 2 different deconfounding approaches. The first deconfounding approach is the traditional linear regression–based approach that removes the contribution of the confounds from the raw brain imaging data. For the second deconfounding approach, we used ComBat to deal with site effects. Similar to the computation of the propensity scores, we performed deconfounding prior to our analyses, based on all the available participants instead of using only the training data. The relationship of performance with diversity after deconfounding is reported using AUC in **Fig 4** and F1 score in **S11 Fig**. We refer to the absence of deconfounding as raw data (first row). In ABIDE, the correlations of AUC and diversity in the holdout dataset when using linear regression–based (*r* = −0.539) and ComBat (*r* = −0.599) deconfounding approaches were slightly lower than the correlation obtained when using the raw data (*r* = −0.671). In HBN, we also found small differences with the linear regression–based (*r* = 0.327) and ComBat (*r* = 0.392) approaches than when using the raw data (*r* = 0.414). Similar results were obtained with F1 score. Note that the computation of the diversity index and the nuisance deconfounding procedures were based on the same set of covariates (i.e., age, sex, and site). Nonetheless, these results show that, although slightly decreased, a strong correspondence persisted between diversity and prediction accuracy after deconfounding. Since diversity captured the variation in the collective covariates, the large proportion of variation in the data that was still explained by diversity (even after deconfounding) suggests that existing deconfounding approaches do not completely eliminate the effects of diversity (i.e., known sources of population variation) from the brain imaging data. It is worth mentioning here that, although there were slight differences, we cannot expect to find major changes because our data were already matched. Nonetheless, these results further emphasized the importance of taking diversity into consideration when using CV strategies to assess the performance of our predictive models.

### Dissecting the impact of diversity on prediction accuracy

**Fig 5:**
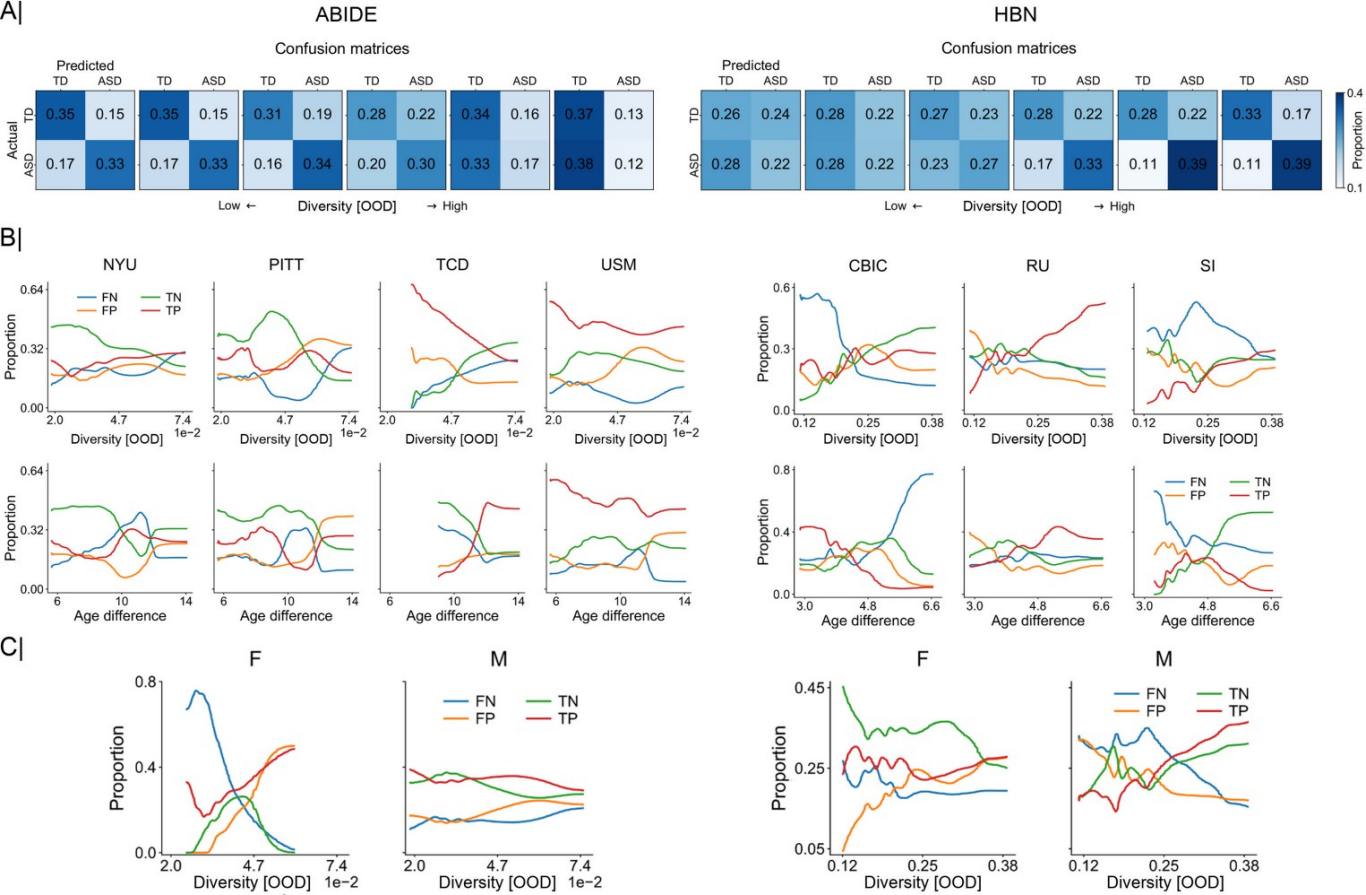
Breakdown of out-of-distribution predictions reveals unstable model behavior according to diversity subdimensions: acquisition sites, participant age, and sex. **(A)** Error matrices that summarize the relationship of diversity (between the training and testing strata, i.e., OOD) with success and failure rate of the predictive models. These matrices are computed across sets of diagnostic classifications sorted in ascending order by diversity. **(B)** Relationship of out-of-distribution performance with diversity and age for each scanning site in ABIDE (left; NYU, PITT, TCD, and USM) and HBN (right; CBIC, RU, and SI). Performance is shown in terms of proportion of FN, FP, TN, and TP cases of model-based classification of clinical diagnoses. **(C)** Relationship of out-of-distribution performance with diversity for female (F) and male (M) participants. Predictive models show unstable behavior across each diversity dimension, underscoring the inconsistent results across the different sites and between females and males. Data underlying this figure can be found in S1 Data. ABIDE, Autism Brain Imaging Data Exchange; CBIC, CitiGroup Corcell Brain Imaging Center; FN, false negatives; FP, false positives; HBN, Healthy Brain Network; NYU, New York University Langone Medical Center; OOD, out of distribution; PITT, University of Pittsburgh, School of Medicine; RU, Rutgers University Brain Imaging Center; SI, Staten Island; TCD, Trinity Centre for Health Sciences, Trinity College Dublin; TN, true negatives; TP, true positives; USM, University of Utah, School of Medicine.

To better understand the impact of diversity on performance, we inspected the out-of-distribution performance of our predictive models using confusion matrices. First, the holdout strata were ranked and divided into 6 chunks according to their diversity (with respect to the training strata) to then generate the respective average confusion matrices. **Fig 5A** shows out-of-distribution confusion matrices in the classification of ASD in both ABIDE and HBN datasets. In ABIDE, with low diversity (e.g., the first confusion matrices) we see that the predictive models achieved good discriminative power, with a proportion of true positives (TP) and true negatives (TN) of 0.33 and 0.35, respectively. But as diversity increased, the model tended to classify most participants as TD. In the confusion matrix corresponding to the highest diversity, the proportion of TN was 0.37 and that of false positives (FP) was 0.38. In the HBN dataset, the impact of diversity was reflected in the opposite direction, starting with low performances (e.g., proportion of TP and TN in the first confusion matrix of 0.22 and 0.25) to then achieve better predictions as diversity increased (e.g., proportion of TP and TN of 0.33 and 0.39 in the last confusion matrix).

We also examined the impact of diversity for each of the covariates that contributed to its computation (i.e., age, sex, and site). As shown in **Fig 5B**, the impact of diversity on classification performance (in terms of true/FP/negatives) varied from one scanning site to another. Considering, for example, the proportion of TN in ABIDE, we can see that it decreased with diversity in most sites except in TCD, where it increased. Similar results could be found in HBN, where the proportion of TN increased with diversity in one single scanning site (i.e., CBIC) but decayed in the remaining sites. This indicates that diversity impacts the performance in each site differently, which might account for the divergent results observed in ABIDE and HBN. When plotting the same performance results against age, we found different trends. This occurred because the distribution of age across the strata was not monotonically increasing with diversity, as shown in **S2 Fig**. Nonetheless, in HBN, e.g., we can see that the proportion of false negatives (FN) increased with age in CBIC, while it decreased in the rest of the scanning sites. Finally, when using sex (see **Fig 5C**), we found a clear decrease in the proportion of FN in females while a slight improvement was observed in males in the ABIDE dataset. Note, however, that there was a substantially reduced number of females in ABIDE compared to HBN. In HBN, we see that the proportion of FP, for example, increased in females and decreased in males. These results indicate that the formed predictive models exhibit unstable performance behavior as a function of population variation, given that prediction accuracy showed varying links with diversity along each dimension. The considered subanalyses need not align with the global trend observed when collapsing all these dimensions of population variation into a single composite index. Furthermore, the divergence in trends between the individual dimensions of interindividual variation (e.g., participant age) and their composite score (i.e., diversity) spotlights the need for propensity score frameworks because they can serve as a handle to monitor and manage diversity in modern multisite datasets in neuroscience. Since we are only interested in the overall trends, the results in **Fig 5B** and **5C** were smoothed using a Gaussian-weighted moving average for visualization purposes.

### Quantifying the impact of diversity on pattern consistency

**Fig 6:**
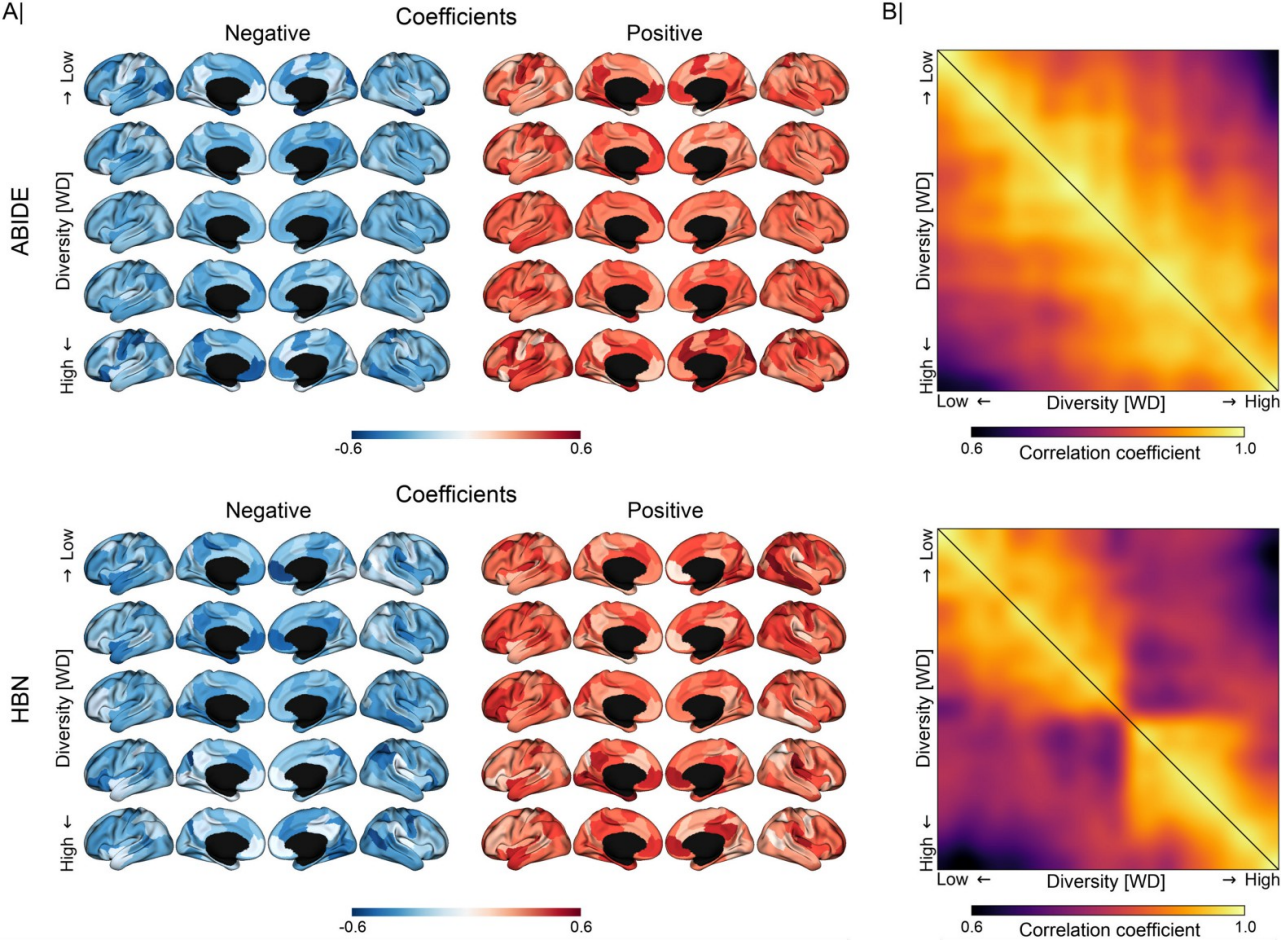
Varying participant diversity is detrimental for consistency of model-derived predictive patterns. Results are based on all possible combinations of 5 out of 10 strata for training and the remaining 5 strata as holdout. **(A)** Drifts in model coefficients when estimated repeatedly (rows) with increasing diversity, separately in ABIDE (top) and HBN (bottom) cohorts. Model coefficients were ranked according to diversity and grouped into 5 chunks. Coefficients averaged within each chunk are displayed with increasing diversity. Positive and negative coefficients are shown in separate brain renderings for visibility. For each node, positive/negative coefficients were computed by averaging the edges with only positive/negative coefficients. **(B)** From top to bottom, changes in predictive model coefficients with increasing diversity in ABIDE and HBN cohorts. Consistency of model coefficients, in terms of Pearson correlation, is obtained for each possible combination of training participants (5 strata combined for training), where diversity is computed as the mean absolute difference in the propensity scores of the training participants (i.e., WD). Each entry in the correlation matrices corresponds to the correlation between the coefficients obtained by 2 predictive models trained on different combinations of training strata. Model coefficients were sorted according to the diversity of their corresponding training observations (arranged from low to high). Each matrix shows correlations based on the model coefficients learned when using the raw data (lower triangular part) and the ComBat-deconfounded data (upper part). Our results show that the consistency of model-derived predictive patterns decays with increasing diversity of the training set, even under deconfounding. Data underlying this figure can be found in S1 Data. ABIDE, Autism Brain Imaging Data Exchange; HBN, Healthy Brain Network; WD, within distribution.

**Fig 7:**
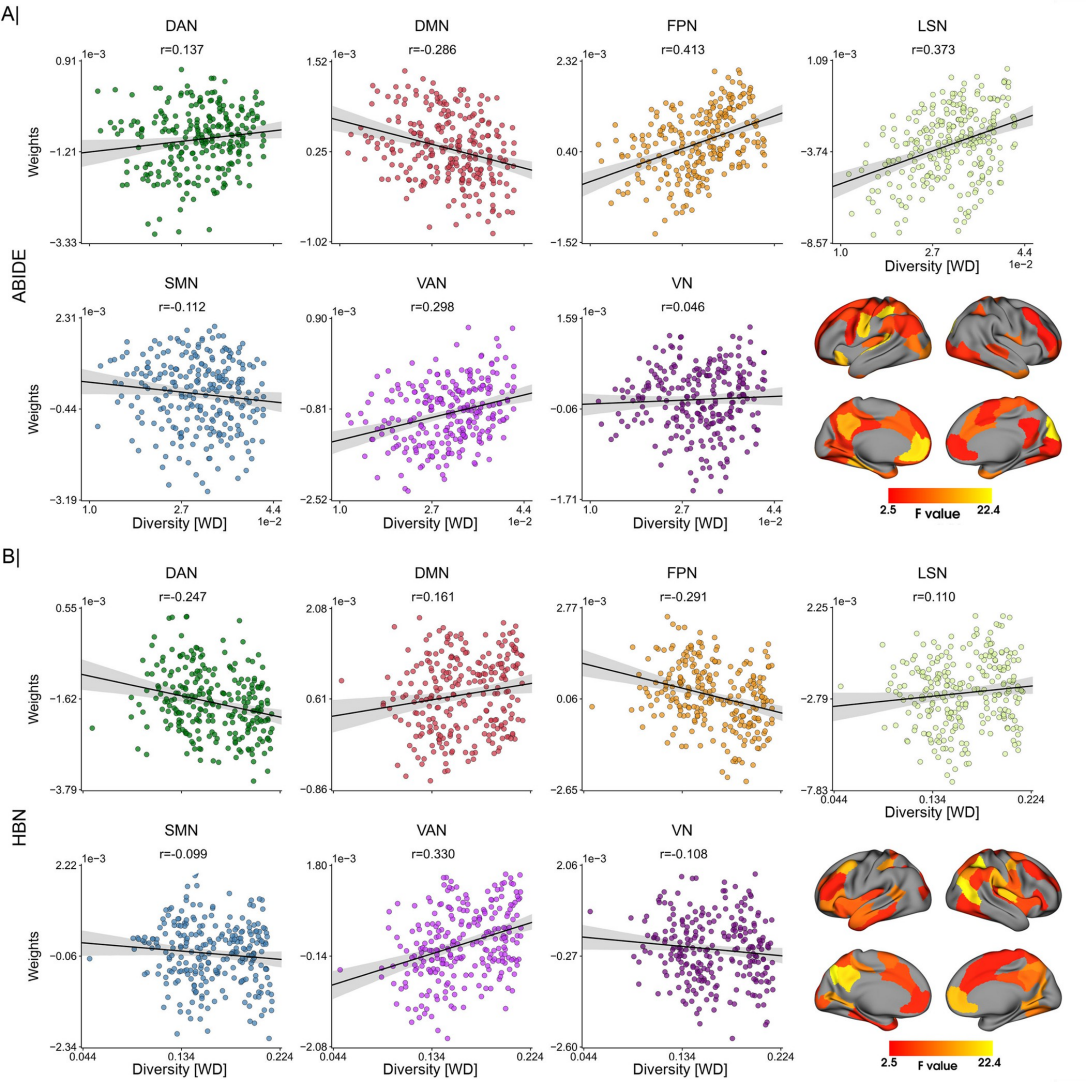
Anatomical hot spots where predictive rules risk to become brittle in the face of diversity. **(A)** ABIDE cohort: relationship between network-aggregated coefficients of predictive model and level of population diversity. Diversity is computed on the training strata (i.e., WD). Model coefficients are averaged for each intrinsic connectivity network. **(B)** HBN cohort: relationship of network-aggregated model coefficients with diversity. Brain renderings in A and B expose regions whose coefficients show a significant association with diversity. Consistent across both clinical cohorts, predictive model coefficients in regions of the highly associative DMN showed a privileged relation to escalating population variation. Data underlying this figure can be found in S1 Data. ABIDE, Autism Brain Imaging Data Exchange; DAN, dorsal attention network; DMN, default mode network; FPN, frontoparietal network; LSN, limbic system network; SMN, somatomotor network; VAN, ventral attention network; VN, visual network; WD, within distribution.

**Fig 6A** displays the coefficients extracted by the predictive models with increasing diversity in both ABIDE and HBN cohorts. Model coefficients were z-scored, grouped into 5 chunks, and averaged according to diversity, with positive and negative coefficients shown separately. In both datasets, we can observe that these coefficients underwent considerable drifts in the face of diversity. For example, the coefficients of both the medial frontal and posterior cingulate cortices changed considerably with diversity, going from positive to negative in ABIDE and vice versa in HBN. Moreover, **Fig 6B** illustrates how consistency changed between patterns extracted from participants in the training set with different diversity. We can see that the extracted patterns gradually diverged as diversity in the training data increased. This is shown in both datasets, but more markedly in HBN. Similar results can be seen in **S12 Fig** in the classification of ASD using cortical thickness and ADHD and ANX based on functional connectivity. The regions whose coefficients underwent significant changes with diversity are shown in **Fig 7A** and **7B** for ABIDE and HBN, respectively. Although this is not directly related to the discriminative importance of such regions, it does show that the patterns extracted from homogeneous observations differ from those obtained when training with more heterogeneous observations. At the network level, we found strong associations of network coefficients with diversity in the default mode (consistency of predictive patterns: *r* = −0.286/0.161 in ABIDE/HBN), frontoparietal (*r* = 0.413/−0.291), limbic (*r* = 0.373/0.110), and dorsal (*r* = 0.137/−0.247) and ventral (*r* = 0.298/0.330) attention networks. There does not seem to be a strong effect of diversity in the model coefficients corresponding to the somatomotor (*r* = −0.112/−0.099) and visual (*r* = 0.046/−0.108) networks.

## Discussion

In our quest toward realizing single-patient prediction in real-world settings, MRI-based machine learning approaches seek to provide accurate and replicable biomarkers. Due to the recent rise of large-scale brain scanning collections, analytical tools are now urgently needed to account for potential distributional shifts as a consequence of increasing diversity of the participant cohorts. In multisite neuroimaging studies, the generalization power of the predictive models is more likely to be affected by several sources of population variation. Some previous research studied their impact on prediction accuracy and biomarker robustness [42,48,89]. Yet, such analyses typically focused on a single source of participant heterogeneity. Our study sought to accommodate cohort diversity coming from multiple sources simultaneously to examine out-of-distribution generalization. We conducted computational experiments that delineate how success and failure of brain-based machine learning predictions are conditioned on sample diversity based on brain imaging–derived profiles of structure and function in 2 different ASD cohorts. Our findings spotlight the heteromodal association cortex, especially the default mode network, as a candidate substrate where participant diversity was linked to the biggest instability of extracted predictive signatures. Our results further show that this interdependence was not only specific to ASD and functional connectivity. Predictive signatures were also driven by participant diversity when studying structural features (i.e., cortical thickness) and functional connectivity in other neuropsychiatric disorders (i.e., ADHD and ANX).

For the purpose of the present work, we construed diversity in terms of 3 key covariates: age, sex, and brain scan acquisition site. These covariates are well known to affect neuroimaging results [43,76,90]. Since ASD is a neurodevelopmental disorder with potential compensatory mechanisms that occur across the life span [91,92], learning an age-independent biomarker from the brain measurements remains a challenging task. From group-level contrast analyses, we know that the form, direction, and magnitude of alterations found in ASD may vary throughout development, as supported by both structural [93–96] and functional [97–99] brain imaging studies. Thus, the complex nature of these disease-related brain correlates hinders the generalizability of machine learning methods across age. These circumstances probably surface as lower classification accuracies that are achieved in participants from different age groups [42,100]. Brain manifestations related to ASD were also found to diverge between males and females [101–103]. In fact, in ASD, there is a well-known prevalence disparity, with an approximate sex ratio of 4 males per female [104]. This observation points to the idea that sex and gender may contribute to diagnosis and that sex differences may interact with autism risk factors [105,106]. Moreover, higher prevalence of males is observed in ADHD [107], while ANX is more common in females [108].

Additionally, when using multisite data, sites may be at odds with respect to other covariates such as age and sex. In our ABIDE cohort, there was only one site that contributed females. Hence, using data from different acquisition sites leads to potential heterogeneity attributable to sampling bias (i.e., demographics) and measurement bias including factors such as scanner type and imaging protocol [35]. In a similar vein, several studies have reported declines in classification performance when using multiple sites [37,42,109]. By designing and executing a framework that jointly considered all these covariates simultaneously, we noticed a considerable drop in out-of-distribution performance in the classification of ASD in ABIDE, based on both functional connectivity and cortical thickness measurements, as well as in patients with ANX in HBN. In contrast, our predictive models achieved higher performance in out-of-distribution classification in patients with ASD and ADHD in HBN. The discrepancy in the impact of diversity on prediction accuracy between ABIDE and HBN may in part be explained by the varying number of females in each dataset (with a considerably higher prevalence in HBN). The width of the propensity score spectrum was also an important factor that differed from one dataset to another. In ABIDE, the propensity score distribution was very narrow, whereas, in HBN, the distribution covered a wider range. The broader the range of propensity scores, the more diverse the individuals in the dataset. In fact, the sex covariate may have contributed substantially to the increased diversity in HBN. Other factors that might have contributed to the different results between ABIDE and HBN include the reduced size of the data in HBN after matching (126 participants in HBN versus 290 in ABIDE), as well as the different sample sizes available from each site. These are important factors to take into consideration when assessing the impact of diversity on prediction accuracy.

In the HBN cohort, our subanalyses focusing on individuals only from one sex showed a similar constellation of findings to those in ABIDE (in both female and male subsets of HBN). However, in the more challenging, yet more realistic setting of multisite datasets, classification accuracy within each site has been shown previously to be highly variable [110], and sites that account for a large portion of the observations may therefore drive prediction performance. Intersite differences also became apparent when we further dissected the role of diversity on performance, where the relationship of performance and diversity varied from one site to another as well as between males and females in both ABIDE and HBN. Overall, our collective findings highlight that diversity had a substantial impact on the achieved prediction accuracy, although inconsistent across the single dimensions of population variation (i.e., age, sex, and scanning site). More importantly, the directionality of the association between diversity and prediction accuracy varied in 2 of the largest open autism brain imaging cohorts available today. In ABIDE, prediction accuracy improved with increasing diversity, whereas it declined in HBN. These insights underscore the need to take into account multiple dimensions of population variation that may potentially contribute to diversity when evaluating the generalizability of a predictive rule, rather than only considering one single factor at a time.

As a central neuroscientific finding from our investigation, the extracted predictive patterns have flagged a coherent set of brain substrates to be associated with participant diversity, which was consistent across ABIDE and HBN cohorts. The robust brain manifestations of diversity included especially regions of the default mode network, as well as the frontoparietal and attention networks. The maturational processes of these associative regions are thought to be more prolonged than those seen in primary sensory or motor brain circuits, which are established earlier in life [111,112]. A more gradual maturation trajectory makes for a wider window for the action of environmental factors that may contribute to the increased functional variability of these neural network systems [113]. Moreover, differences in myelin content are also observed between these canonical networks, with the higher association cortex showing fewer myelinated axons than primary sensory and motor cortices. Less myelination might enable greater plasticity in association areas both during development and adulthood [114–116]. Neural circuits with less myelinated regions have previously been shown to exhibit increased functional and structural variability [117]. Taken together, these factors might be related to, at least partly, the high susceptibility of our predictive models to population diversity in the default mode network, and other associative cortical regions.

More specifically, in ASD, these functional systems commonly subsumed as uni- and heteromodal association cortex have frequently been reported to be disrupted in a long series of existing studies [97,118–124]. Additionally, regions within these networks were found to be discriminative in machine learning classification of ASD versus TD participants [9,39,125]. The high variability in the coefficients of these networks in relation with our diversity indicators is in line with the inconsistencies regarding hypo/hyperconnectivity findings in ASD. In the default mode, for example, the direction of the disease-related functional connectivity varies with the age of the cohort under study, shifting from hyperconnectivity in children to hypoconnectivity in adults, with both co-occurring in cohorts with broader age ranges [126]. In addition, these networks are highly idiosyncratic in ASD, showing greater topographic variability among ASD individuals than TD controls [127–129]. This increased idiosyncrasy may also contribute to this elusiveness of default network and other association regions to yield stable predictive signatures. Hence, our collective findings make apparent that diversity selectively impacts pattern stability especially for some of the highest integration centers of the human cerebral cortex, which we note to coincide with the functional systems that are, instead, routinely reported to be specific for patients with ASD.

The proof-of-principle analyses in this work were centered on age, sex, and site because these indicators of diversity are among the frequently available in many biomedical datasets. However, there are several factors that could and should be considered in future research: verbal and nonverbal IQ, open versus closed eyes during functional brain scanning, ethnicity/race, and comorbidity among others. Comorbidities, for example, may play an important role in prediction accuracy because participants with the same comorbidity may have more similar biomarkers. In HBN, when classifying TD versus ASD, e.g., if ASD individuals in both train and test sets shared the same comorbidities, we would expect the predictive models to achieve better performance than when trained on data with no comorbidities. Thus, predictive models built based on participants with other comorbidities would likely have better generalization abilities. Including additional comorbidity covariates (i.e., ADHD and ANX) in the propensity score model can allow us to accommodate more sources of diversity to better understand the differences in prediction accuracy. Nonetheless, note that in binary classification problems involving healthy controls (e.g., TD versus ASD), comorbidities are only present in one group. Incorporating comorbidities may be therefore more suitable for classification problems where the comorbidities are present in both groups (e.g., ASD versus ADHD, with ANX as the comorbidity). Still, all these factors may play a pivotal role for classification accuracy and pattern stability of predictive models, from support vector classification to random forest algorithms to deep artificial neural networks. By benefitting from propensity scores, investigators can integrate the covariates or dimensions of population variation into the analytical workflow to carefully monitor their implications for a statistical analysis of interest. Additionally, our study only considered the role of diversity in classification, but our analysis pipelines are also applicable to regression problems. In the latter, diversity can be computed based on generalized propensity scores [130,131], which are an extension of propensity scores to work with continuous variables [132]. Propensity scores could be used, e.g., in the prediction of symptom severity (e.g., ADOS) rather than diagnosis. Indeed, focusing on symptom severity instead of diagnosis may account for part of the heterogeneity observed in ASD [133].

The generalization failure exposed by the present analyses highlights an important instance of dataset shift [134], a well-established concept in the machine learning community. Dataset shift occurs when the joint distribution of the test data differs from that of the training data. There are different types of dataset shift. In order to determine the type of shift we need to identify (i) the factors of the joint distribution that change between the training and testing stages of the predictive modeling workflow; and (ii) the causal relationship between outcome variable and the brain features [135,136]. Thus, elucidating the causal structure of the variables at play in our problem would help identify susceptibility to shifts and build more robust predictive models [137]. This realization highlights the importance of future work to exploit causal knowledge to better deal with potential distributional shifts. For example, incorporating causal knowledge has been shown to allow for better performance than the traditional linear regression–based approach for deconfounding [138]. In our case, repeating our analysis using different linear deconfounding routines only led to slight differences in the relationship between model performance and diversity. This observation underlines the limitations of the deconfounding approaches that the neuroimaging community is employing nowadays. Moreover, in our study, we analyzed dataset distribution shift by focusing on the shift as captured by the propensity scores of known sources of population variation (i.e., covariates of the participants) from the training to the testing stage. As such, a difference in propensity scores is a good indicator of dataset shift only when the shift in covariates is also reflected in a similar shift in the brain imaging features. When changes in propensity scores are manifested in shifts in brain imaging features, prediction performance is likely to be influenced by participant diversity. It is important to note here that these distribution shifts in brain imaging features might be class specific (different in TD than in ASD participants). For example, functional connectivity in ASD shifts with age from hyperconnectivity in children to hypoconnectivity in adults [126]. In our experiments, model accuracy decreased with increasing diversity in ABIDE (both with functional connectivity and cortical thickness), and in HBN (functional connectivity) when studying males or females separately, but not when including both males and females in the analysis. The divergent results between ABIDE and HBN indicate that a shift in propensity scores is not reflected in similar changes in the brain imaging features in ABIDE and HBN. This is to be expected because sex had an important contribution in the estimating the propensity scores in HBN, whereas its contribution in ABIDE was negligible. Furthermore, this may occur because differences in propensity scores do not always represent a shift in the brain imaging features, which may be due to unmeasured covariates or sources of population variation.

Adjusting for the “right” confounding covariates is a causal matter at its heart. Our study goal has a direct relationship to the notion of ignorability from the causal inference literature—a form of independence that is sometimes also referred to as unconfoundedness [44,139–141]. Put simply, this concept encapsulates the hope of the investigator that all relevant sources of variation have been adequately captured by the set of variables that are available for quantitative analysis. That is, assuming ignorability carries the bet by the analyst that all confounding covariates of no scientific interest are known and have been measured and that there is no external knowledge outside of the data that would allow further characterizing the participant assessments: $y_{diagnosis}⊥X_{context\;of\;brain\;scan\;acquisition}|C$. In everyday research practice, this is an exceptionally strong assumption about the process that gave rise to the dataset at hand. This is because, if ignorability is truly satisfied, given the available covariates, the investigator is able to infer causal effects from the data without bias or skewing of the findings. In our present examination, however, propensity score–matched participants varied widely in the predictive performance and the extracted decision rules for classification. After repeated pulls of different population strata, the behavior of our predictive models diverged considerably when queried to distinguish participants with a neuropsychiatric diagnosis and neurotypical controls. Even after deconfounding for participant age, sex at birth, and data acquisition site, our pairs of brain feature profile and target diagnosis were not mutually exchangeable and would also violate the independent and identically distributed (i.i.d.) assumption (exchangeability being a more lenient or more general assumption). Therefore, although there were some differences in the participant composition of ABIDE and HBN that might have contributed to the diverging results in out-of-distribution prediction scaling as a function of diversity in these datasets (e.g., differences in sample size, proportion of females, and range of propensity score distribution), this divergence may be due to potentially many other sources of population variation. There are certain sources of population variation that are known, measured, and that we can explicitly model (e.g., age, sex, and scanning site in our case). In contrast, others are not included in the propensity score model (e.g., comorbidity only available in HBN), are unavailable (e.g., ethnicity/race information), or are even unknown at present (so-called “unknown unknowns”). These unobserved sources of population variation are the most likely culprit behind the diverging model performance behaviors between ABIDE and HBN. With unobserved variables, the ignorability assumption does probably not hold, and hence our diversity index (based solely on 3 dimensions of population variation, e.g., age, sex, and site) can, by construction, only seize the variation of the known sources of population variation. Yet, the remaining untracked diversity is likely to have uncontrollable effects and its cost is what is exposed by the diverging results between HBN and ABIDE. This observation would be compatible with the more general conclusion that the ignorability assumption is not admissible in many or most brain imaging studies given the types of confounding covariates that are measured in common large-scale datasets. It is plausible that there are lurking confounding variables, possibly attributable to latent population structure, which we are not yet in the habit of collecting or accounting for [142].

We admit several limitations about our quantitative findings and substantive conclusions. First, participants were matched using propensity scores prior to conducting further steps of the analysis workflow. This decision resulted in smaller datasets for learning our predictive models, especially in the HBN cohort. To maximize the participants available for model estimation, we used a caliper of 0.2 that is commonly used in the literature [74,75]. This, in turn, conceded to imperfect matches in certain cases. Smaller caliper values, such as 0.1, may have decreased the proportion of matches, but provided paired participants with more similar covariates. Moreover, during stratification, participants were grouped in equally sized rather than strata that were equally spaced in the diversity spectrum. We used equally sized strata to control the number of participants per strata. However, the covered diversity range may be different from one stratum to another, which may have impacted the computations. Finally, in our experiments, we found trends with different directionality when studying the impact of diversity on out-of-distribution performance in ABIDE and HBN. However, similar trends were found when only considering males or females in HBN. Given the differences between ABIDE and HBN datasets, future work may involve investigating the impact of diversity on prediction accuracy using additional large-scale multisite neuroimaging datasets (e.g., [143]) to gain more insight into this relationship. Nonetheless, our study showcases that sources of population variation play an important role in predictions from brain imaging to a degree that is currently underappreciated, independent of the directionality.

To conclude, we have added a sharp instrument to the analytical toolkit of neuroscience investigators. Diversity-aware machine learning pipelines allow tracking, unpacking, and understanding out-of-distribution generalization in a variety of research agendas, such as studies involving brain scans. By bringing propensity scores to the neuroimaging community, participant stratification and cohort homogenization can be carried out in a seamless fashion with few or potentially many dozen covariates, be they categorical or continuous in nature. This principled solution can effectively detect and handle pivotal sources of demographic, clinical, or technical variation, which can hurt the development of predictive models. Moreover, wider adoption of propensity scores can open new avenues to explore causal underpinnings of how brain representations are linked to the repertoire of human behavior. Based on our collective findings across 2 confederated neuroimaging efforts and different psychiatric conditions, participant diversity is a key factor contributing to generalization performance. Furthermore, the robustness of the predictive biomarkers cannot be guaranteed by the deconfounding practices that are the norm today.

## Supporting information

S1 Table Scanner and data acquisition settings. CBIC, CitiGroup Corcell Brain Imaging Center; FA, flip angle; NYU, New York University Langone Medical Center; PITT, University of Pittsburgh, School of Medicine; RU, Rutgers University Brain Imaging Center; SI, Staten Island; TCD, Trinity Centre for Health Sciences, Trinity College Dublin; TE, echo time; TI, inversion time; TR, repetition time; USM, University of Utah, School of Medicine.
(DOCX)

S2 Table Demographics for each site and group after participant matching. Number of participants (*N*), males/females (M/F), and mean age and standard deviation for each site and group. Note that matching was performed separately for each of the different psychiatric conditions (i.e., COND) in HBN. CBIC, CitiGroup Corcell Brain Imaging Center; HBN, Healthy Brain Network; NYU, New York University Langone Medical Center; PITT, University of Pittsburgh, School of Medicine; RU, Rutgers University Brain Imaging Center; SI, Staten Island; TCD, Trinity Centre for Health Sciences, Trinity College Dublin; USM, University of Utah, School of Medicine.
(DOCX)

S1 Fig Absolute SMDs in covariates between groups. SMDs are computed between TD and ASD participants in the original (i.e., unmatched) and the matched data. Results are reported for both ABIDE and HBN. For each dataset, balance was assessed for age, sex, and the different scanning sites. Data underlying this figure can be found in S1 Data. ABIDE, Autism Brain Imaging Data Exchange; ASD, autism spectrum disorder; CBIC, CitiGroup Corcell Brain Imaging Center; HBN, Healthy Brain Network; NYU, New York University Langone Medical Center; PITT, University of Pittsburgh, School of Medicine; RU, Rutgers University Brain Imaging Center; SI, Staten Island; SMD, standardized mean difference; TCD, Trinity Centre for Health Sciences, Trinity College Dublin; TD, typically developing; USM, University of Utah, School of Medicine.
(TIF)

S2 Fig Distribution of diversity indicators across strata. From left to right, distribution of age, sex, and site across strata in ABIDE (top) and HBN (bottom) datasets. The fact that, in HBN, we have females at only one extreme of the propensity score spectrum (i.e., in the strata with the lowest propensity scores) corroborates the validity of our approach to characterize diversity. Data underlying this figure can be found in S1 Data. ABIDE, Autism Brain Imaging Data Exchange; HBN, Healthy Brain Network.
(TIF)

S3 Fig Comparison of covariates versus brain imaging data in the classification of ASD before and after matching. Results are based on functional connectivity and reported using original (i.e., unmatched) and matched data in both ABIDE and HBN. Performance is measured using AUC based on a conventional 10-fold CV scheme. While the covariates (i.e., age, sex, and scanning site) showed good predictive power in discriminating between ASD and TD before matching (especially in HBN), with the matching procedure the covariates were no longer informative in either dataset. Data underlying this figure can be found in S1 Data. ABIDE, Autism Brain Imaging Data Exchange; ASD, autism spectrum disorder; AUC, area under the ROC curve; CV, cross-validation; HBN, Healthy Brain Network; TD, typically developing.
(TIF)

S4 Fig Sex contribution to diversity negligible in ABIDE and strong in HBN. Pearson correlation (*r*) between diversity (based on the difference in propensity scores between training and test data (i.e., OOD) and the difference in the proportion of females. These plots show that sex had a strong contribution to diversity, while it had almost no contribution in ABIDE. Data underlying this figure can be found in S1 Data. ABIDE, Autism Brain Imaging Data Exchange; HBN, Healthy Brain Network; OOD, out of distribution.
(TIF)

S5 Fig Out-of-distribution prediction and performance of predictive models as a function of sample diversity. Left: comparison of model performance based on contiguous (Cont) and diverse (Div) training sets in the classification of ASD (top) using CT in ABIDE and ADHD (middle) and ANX (bottom) based on functional connectivity in HBN. Performance is assessed using different sizes of training data (from 2 to 8 combined strata). For each disorder, the first column reports the performance using a 10-fold CV strategy based solely on the training data, whereas the second column displays the performance in the holdout set, which contains the remaining participants (from untouched strata). An additional model (i.e., Rand) is used as a baseline, where training participants are randomly chosen regardless of their diversity (propensity scores). Right: prediction performance based on all possible combinations of 5 out of 10 strata for training (and the remaining 5 for holdout). The first column reports the predictive model performance using a 10-fold CV strategy based solely on the training set, where diversity is computed as the average of all pairwise absolute differences in propensity scores (i.e., WD). The second column displays the performance for each single stratum in the holdout dataset, and diversity denotes the mean absolute difference in propensity scores between the participants of the training set and those in the held-out participants (i.e., OOD). The strength of the association between performance and diversity is reported with Pearson correlation coefficient (*r*). Data underlying this figure can be found in S1 Data. ABIDE, Autism Brain Imaging Data Exchange; ADHD, attention-deficit/hyperactivity disorder; ASD, autism spectrum disorder; CT, cortical thickness; CV, cross-validation; HBN, Healthy Brain Network; OOD, out of distribution; WD, within distribution.
(TIF)

S6 Fig Consistency of extracted predictive patterns. Consistency of model coefficients, as quantified by Pearson correlation coefficient, obtained when using contiguous (Cont), diverse (Div), and random (Rand) sampling of participants in the classification of autism using CT in the ABIDE dataset (left), and in the classification of ADHD (middle) and ANX (right) based on functional connectivity in the HBN dataset. For each of these sampling schemes, consistency is shown for different numbers of combined strata used for predictive model training (from 2 to 8 combined strata), delineated by the green segments. Data underlying this figure can be found in S1 Data. ABIDE, Autism Brain Imaging Data Exchange; ADHD, attention-deficit/hyperactivity disorder; ANX, anxiety; CT, cortical thickness; HBN, Healthy Brain Network.
(TIF)

S7 Fig Relationship between performance of predictive models and diversity in males and females (separately) in the HBN cohort. Diversity is computed based on age and scanning site. After matching, analysis was carried out on 82 males and 44 females. Due to the small size of the data, we used all possible combinations of 4 out of 8 (instead of 5 out of 10) strata for training and the remaining 4 strata as holdout. Prediction accuracy based on AUC (top) and F1 score (bottom) in 2 subsets of the HBN dataset: only males (left) and only females (right). For each subset, the first column indicates the predictive model performance using a 10-fold CV strategy based solely on the training set, where diversity is computed as the average of all pairwise absolute differences in propensity scores (i.e., WD). The second column displays the performance for each single stratum in the holdout strata. Diversity denotes the mean absolute difference in propensity scores between the participants of the training set and those in the held-out strata with unseen participants (i.e., OOD). The strength of the association between performance and diversity is reported with Pearson correlation coefficient (*r*). Data underlying this figure can be found in S1 Data. AUC, area under the curve; CV, cross-validation; HBN, Healthy Brain Network; OOD, out of distribution; WD, within distribution.
(TIF)

S8 Fig Random versus diversity-aware sampling comparison. We repeated the out-of-distribution analysis in Fig 3 using the same number of draws but ignoring the strata (i.e., Random sampling) and compared the results to the original sampling strategy based on stratification (i.e., Diversity-aware sampling). Results are shown for ABIDE (top) and HBN (bottom) for both AUC and F1 score performance metrics. Left: distributions of diversity between the train and test subsets for each draw (i.e., data split) based on random (blue) and our diversity-aware sampling (orange). Relationship of diversity with OOD prediction performance when using random (middle) and diversity-aware sampling (right). Ignoring the strata (i.e., random sampling) produces train–test splits with practically no difference in their covariates, which, in turn, does not allow us to analyze the impact of diversity in performance. On the other hand, with stratification, we were able to perform a diversity-aware sampling that produces train–test splits with different covariates. Data underlying this figure can be found in S1 Data. ABIDE, Autism Brain Imaging Data Exchange; AUC, area under the curve; HBN, Healthy Brain Network; OOD, out of distribution.
(TIF)

S9 Fig Out-of-distribution classification performance versus diversity before and after matching. **(A)** From top to bottom, proportion of ASD individuals in each stratum, AUC versus diversity, and F1 score versus diversity. For each dataset (ABIDE and HBN), the first column shows results based on unmatched data, while the results based on the matched data are shown in the second column. **(B)** Relationship between out-of-distribution performance (both AUC and F1 score) with the proportion of ASD individuals in each stratum based on unmatched data. From these results, we see that there is a high imbalance in strata when using unmatched data, especially in HBN. The proportion of patients with ASD is lower/higher in the strata with the lowest/highest propensity scores in HBN. Regarding performance, there were no important differences in the relationship of prediction accuracy with diversity when using the matched or unmatched data in most scenarios, with the exception of F1 score in HBN. The correlation of F1 score with diversity in HBN was negative when using unmatched data (*r* = −0.312) and positive when using the matched data (*r* = 0.297). This occurs because the F1 score is highly affected by class imbalance (as shown in B). When using unmatched data, it is difficult to tell if prediction accuracy is related to the diversity in the data, to the imbalance in the strata, or even to both. Matching is thus required to prevent class imbalance from biasing the results. Data underlying this figure can be found in S1 Data. ABIDE, Autism Brain Imaging Data Exchange; ASD, autism spectrum disorder; HBN, Healthy Brain Network.
(TIF)

S10 Fig Example of a group-wise feature difference in ABIDE and HBN scenarios. We generated a dataset based on a single feature to illustrate the divergent results between ABIDE and HBN. **(A)** Possible scenarios explaining the shift in our feature (x) between training and testing stages. In ABIDE, a drop in performance may occur when the pattern learned by the predictive model during training is different from the one in the test data. In HBN, on the other hand, it is easier for the predictive models to discriminate between groups in the test set, which may explain the increase in performance with diversity. **(B)** We generated a dataset where the changes in group-wise feature difference (Δ x) are a function of propensity scores. In ABIDE, the lower/higher the propensity scores the more difficult to discriminate between groups, whereas in HBN (Δx) increases as we move toward the extremes of the propensity score spectrum, thus improving the discriminative power. **(C)** Based on these data, we assessed the relationship between diversity and AUC. From the results, we can see that these scenarios explain the divergent results obtained in ABIDE and HBN. Data underlying this figure can be found in S1 Data. ABIDE, Autism Brain Imaging Data Exchange; AUC, area under the curve; HBN, Healthy Brain Network.
(TIF)

S11 Fig Established and new deconfounding strategies appear insufficient to counteract escalating population diversity. A means to quantify the behavior of predictive models is comparing its predictions on training and testing information. Performance is measured by F1 score on the original brain imaging data (Raw, top), and after carrying out deconfounding steps on the brain feature prior to the pattern classification pipeline using standard linear regression–based deconfounding (LinReg, middle) and recently proposed ComBat (bottom) approaches. Compared with the conventional nuisance removal using regression residuals, ComBat is a more advanced hierarchical regression to control for site differences. Results are reported for 2 different clinical cohorts: ABIDE (left) and HBN (right). For each cohort, the first column shows the model prediction performance using a 10-fold CV strategy. Diversity is computed as the average of pairwise absolute differences in propensity scores between all participants (i.e., WD). Each dot is a cross-validated accuracy. The second column, for each cohort, displays the performance in the holdout participants. Instead of reporting performance on all the participants in the holdout data, here performance is assessed independently for participants in each single stratum from the holdout data. Thus, diversity denotes the mean absolute difference in propensity scores between the participants in the training set and those in the held-out stratum (i.e., OOD). Data underlying this figure can be found in S1 Data. ABIDE, Autism Brain Imaging Data Exchange; CV, cross-validation; HBN, Healthy Brain Network; OOD, out of distribution; WD, within distribution.
(TIF)

S12 Fig Consistency of model-derived predictive patterns as a function of sample diversity. Changes in predictive model coefficients with increasing diversity in the classification of autism using CT in the ABIDE dataset (left), and in the classification of ADHD (middle) and ANX (right) based on functional connectivity in the HBN dataset. Consistency of model coefficients, in terms of Pearson correlation, is obtained for each possible combination of training set (5 strata combined for training), where diversity is computed as the mean absolute difference in the propensity scores of the training observations (i.e., WD). Pattern correlations are sorted according to their corresponding diversity (from low to high) based on the raw data (lower triangular part) and the ComBat-deconfounded data (upper part). Pearson correlation is based on the average of 10 consecutive model coefficients, according to their diversity. Data underlying this figure can be found in S1 Data. ABIDE, Autism Brain Imaging Data Exchange; ADHD, attention-deficit/hyperactivity disorder; ANX, anxiety; CT, cortical thickness; HBN, Healthy Brain Network; WD, within distribution.
(TIF)

S1 Data Data underlying figures. (XLSX)

## Abbreviations

ABIDE: Autism Brain Imaging Data Exchange
ADHD: attention-deficit/hyperactivity disorder
ADI-R: Autism Diagnostic Interview-Revised
ADOS: Autism Diagnostic Observation Schedule
ANOVA: analysis of variance
ANX: anxiety
ASD: autism spectrum disorder
AUC: area under the receiver operating characteristic curve
CBIC: CitiGroup Corcell Brain Imaging Center
CV: cross-validation
FN: false negatives
FWHM: full width at half maximum
HBN: Healthy Brain Network
i.i.d.: independent and identically distributed
NYU: New York University Langone Medical Center
PITT: University of Pittsburgh, School of Medicine
rs-fMRI: resting-state functional MRI
RU: Rutgers University Brain Imaging Center
SI: Staten Island
SMD: standardized mean difference
TCD: Trinity Centre for Health Sciences, Trinity College Dublin
TD: typically developing
TN: true negatives
TP: true positives
T1w: T1-weighted
USM: University of Utah, School of Medicine

## References

[1] Gabrieli JD, Ghosh SS, Whitfield-Gabrieli S. Prediction as a humanitarian and pragmatic contribution from human cognitive neuroscience. Neuron. 2015;85(1):11–26. doi: 10.1016/j.neuron.2014.10.047 25569345 PMC4287988

[2] Bzdok D. Classical statistics and statistical learning in imaging neuroscience. Front Neurosci. 2017;11:543. doi: 10.3389/fnins.2017.00543 29056896 PMC5635056

[3] Bzdok D, Yeo BT. Inference in the age of big data: Future perspectives on neuroscience. Neuroimage. 2017;155:549–64. doi: 10.1016/j.neuroimage.2017.04.061 28456584

[4] Orru G, Pettersson-Yeo W, Marquand AF, Sartori G, Mechelli A. Using support vector machine to identify imaging biomarkers of neurological and psychiatric disease: a critical review. Neurosci Biobehav Rev. 2012;36(4):1140–52. doi: 10.1016/j.neubiorev.2012.01.004 22305994

[5] Pereira F, Mitchell T, Botvinick M. Machine learning classifiers and fMRI: a tutorial overview. Neuroimage. 2009;45(1):S199–209. doi: 10.1016/j.neuroimage.2008.11.007 19070668 PMC2892746

[6] Brammer M. The role of neuroimaging in diagnosis and personalized medicine-current position and likely future directions. Dialogues Clin Neurosci. 2009;11(4):389. doi: 10.31887/DCNS.2009.11.4/mbrammer 20135896 PMC3181933

[7] Bzdok D, Varoquaux G, Steyerberg EW. Prediction, Not Association, Paves the Road to Precision Medicine. JAMA Psychiat. 2021 Feb 1;78(2):127–8. 10.1001/jamapsychiatry.2020.2549 32804995

[8] Heinsfeld AS, Franco AR, Craddock RC, Buchweitz A, Meneguzzi F. Identification of autism spectrum disorder using deep learning and the ABIDE dataset. Neuroimage Clin. 2018;17:16–23. doi: 10.1016/j.nicl.2017.08.017 29034163 PMC5635344

[9] Plitt M, Barnes KA, Martin A. Functional connectivity classification of autism identifies highly predictive brain features but falls short of biomarker standards. Neuroimage Clin. 2015;7:359–66. doi: 10.1016/j.nicl.2014.12.013 25685703 PMC4309950

[10] Sabuncu MR, Konukoglu E. Alzheimer’s Disease Neuroimaging Initiative. Clinical prediction from structural brain MRI scans: a large-scale empirical study. Neuroinformatics. 2015;13(1):31–46. doi: 10.1007/s12021-014-9238-1 25048627 PMC4303550

[11] Wolfers T, Floris DL, Dinga R, van Rooij D, Isakoglou C, Kia SM, et al. From pattern classification to stratification: towards conceptualizing the heterogeneity of Autism Spectrum Disorder. Neurosci Biobehav Rev. 2019;104:240–54. doi: 10.1016/j.neubiorev.2019.07.010 31330196

[12] Brown MR, Sidhu GS, Greiner R, Asgarian N, Bastani M, Silverstone PH, et al. ADHD-200 Global Competition: diagnosing ADHD using personal characteristic data can outperform resting state fMRI measurements. Front Syst Neurosci. 2012;6:69. doi: 10.3389/fnsys.2012.00069 23060754 PMC3460316

[13] Riaz A, Asad M, Alonso E, Slabaugh G. DeepFMRI: End-to-end deep learning for functional connectivity and classification of ADHD using fMRI. J Neurosci Methods. 2020;335:108506. doi: 10.1016/j.jneumeth.2019.108506 32001294

[14] Sen B, Borle NC, Greiner R, Brown MR. A general prediction model for the detection of ADHD and Autism using structural and functional MRI. PLoS ONE. 2018;13(4):e0194856. doi: 10.1371/journal.pone.0194856 29664902 PMC5903601

[15] Wang X-H, Jiao Y, Li L. Identifying individuals with attention deficit hyperactivity disorder based on temporal variability of dynamic functional connectivity. Sci Rep. 2018;8(1):1–12. doi: 10.1038/s41598-017-17765-5 30087369 PMC6081414

[16] Frick A, Gingnell M, Marquand AF, Howner K, Fischer H, Kristiansson M, et al. Classifying social anxiety disorder using multivoxel pattern analyses of brain function and structure. Behav Brain Res. 2014;259:330–5. doi: 10.1016/j.bbr.2013.11.003 24239689 PMC3888925

[17] Liu F, Guo W, Fouche J-P, Wang Y, Wang W, Ding J, et al. Multivariate classification of social anxiety disorder using whole brain functional connectivity. Brain Struct Funct. 2015;220(1):101–15. doi: 10.1007/s00429-013-0641-4 24072164

[18] Davatzikos C, Shen D, Gur RC, Wu X, Liu D, Fan Y, et al. Whole-brain morphometric study of schizophrenia revealing a spatially complex set of focal abnormalities. Arch Gen Psychiatry. 2005;62(11):1218–27. doi: 10.1001/archpsyc.62.11.1218 16275809

[19] Rozycki M, Satterthwaite TD, Koutsouleris N, Erus G, Doshi J, Wolf DH, et al. Multisite machine learning analysis provides a robust structural imaging signature of schizophrenia detectable across diverse patient populations and within individuals. Schizophr Bull. 2018;44(5):1035–44. doi: 10.1093/schbul/sbx137 29186619 PMC6101559

[20] Shen H, Wang L, Liu Y, Hu D. Discriminative analysis of resting-state functional connectivity patterns of schizophrenia using low dimensional embedding of fMRI. Neuroimage. 2010;49(4):3110–21. doi: 10.1016/j.neuroimage.2009.11.011 19931396

[21] Yassin W, Nakatani H, Zhu Y, Kojima M, Owada K, Kuwabara H, et al. Machine-learning classification using neuroimaging data in schizophrenia, autism, ultra-high risk and first-episode psychosis. Transl Psychiatry. 2020;10(1):1–11. doi: 10.1038/s41398-019-0665-5 32801298 PMC7429957

[22] Karrer TM, Bassett DS, Derntl B, Gruber O, Aleman A, Jardri R, et al. Brain-based ranking of cognitive domains to predict schizophrenia. Hum Brain Mapp. 2019;40(15):4487–507. doi: 10.1002/hbm.24716 31313451 PMC6865423

[23] Arbabshirani MR, Plis S, Sui J, Calhoun VD. Single subject prediction of brain disorders in neuroimaging: Promises and pitfalls. Neuroimage. 2017;145:137–65. doi: 10.1016/j.neuroimage.2016.02.079 27012503 PMC5031516

[24] Pulini AA, Kerr WT, Loo SK, Lenartowicz A. Classification accuracy of neuroimaging biomarkers in attention-deficit/hyperactivity disorder: effects of sample size and circular analysis. Biol Psychiatry Cogn Neurosci Neuroimaging. 2019;4(2):108–20. doi: 10.1016/j.bpsc.2018.06.003 30064848 PMC6310118

[25] Woo C-W, Chang LJ, Lindquist MA, Wager TD. Building better biomarkers: brain models in translational neuroimaging. Nat Neurosci. 2017 Feb 23;20(3):365–77. doi: 10.1038/nn.4478 28230847 PMC5988350

[26] Alexander LM, Escalera J, Ai L, Andreotti C, Febre K, Mangone A, et al. An open resource for transdiagnostic research in pediatric mental health and learning disorders. Scientific Data. 2017;4(1):1–26. doi: 10.1038/sdata.2017.181 29257126 PMC5735921

[27] Biswal BB, Mennes M, Zuo X-N, Gohel S, Kelly C, Smith SM, et al. Toward discovery science of human brain function. Proc Natl Acad Sci U S A. 2010;107(10):4734–9. doi: 10.1073/pnas.0911855107 20176931 PMC2842060

[28] Casey BJ, Cannonier T, Conley MI, Cohen AO, Barch DM, Heitzeg MM, et al. The adolescent brain cognitive development (ABCD) study: imaging acquisition across 21 sites. Dev Cogn Neurosci. 2018;32:43–54. doi: 10.1016/j.dcn.2018.03.001 29567376 PMC5999559

[29] Di Martino A, Yan C-G, Li Q, Denio E, Castellanos FX, Alaerts K, et al. The autism brain imaging data exchange: towards a large-scale evaluation of the intrinsic brain architecture in autism. Mol Psychiatry. 2014;19(6):659–67. doi: 10.1038/mp.2013.78 23774715 PMC4162310

[30] Di Martino A, O’connor D, Chen B, Alaerts K, Anderson JS, Assaf M, et al. Enhancing studies of the connectome in autism using the autism brain imaging data exchange II. Sci Data. 2017;4(1):1–15. doi: 10.1038/sdata.2017.10 28291247 PMC5349246

[31] Van Essen DC, Smith SM, Barch DM, Behrens TE, Yacoub E, Ugurbil K, et al. The WU-Minn human connectome project: an overview. Neuroimage. 2013;80:62–79. doi: 10.1016/j.neuroimage.2013.05.041 23684880 PMC3724347

[32] Milham MP, Craddock RC, Son JJ, Fleischmann M, Clucas J, Xu H, et al. Assessment of the impact of shared brain imaging data on the scientific literature. Nat Commun. 2018;9(1):1–7. doi: 10.1038/s41467-017-02088-w 30026557 PMC6053414

[33] Poldrack R, Baker C, Durnez J, Gorgolewski K, Matthews P, Munafò M, et al. Scanning the horizon: towards transparent and reproducible neuroimaging research. Nat Rev Neurosci. 2017;18(2):115–26. doi: 10.1038/nrn.2016.167 28053326 PMC6910649

[34] Kostro D, Abdulkadir A, Durr A, Roos R, Leavitt BR, Johnson H, et al. Correction of inter-scanner and within-subject variance in structural MRI based automated diagnosing. Neuroimage. 2014;98:405–15. doi: 10.1016/j.neuroimage.2014.04.057 24791746

[35] Yamashita A, Yahata N, Itahashi T, Lisi G, Yamada T, Ichikawa N, et al. Harmonization of resting-state functional MRI data across multiple imaging sites via the separation of site differences into sampling bias and measurement bias. PLoS Biol. 2019;17(4):e3000042. doi: 10.1371/journal.pbio.3000042 30998673 PMC6472734

[36] Chen H, Duan X, Liu F, Lu F, Ma X, Zhang Y, et al. Multivariate classification of autism spectrum disorder using frequency-specific resting-state functional connectivity—a multi-center study. Prog Neuropsychopharmacol Biol Psychiatry. 2016;64:1–9. doi: 10.1016/j.pnpbp.2015.06.014 26148789

[37] Nielsen JA, Zielinski BA, Fletcher PT, Alexander AL, Lange N, Bigler ED, et al. Multisite functional connectivity MRI classification of autism: ABIDE results. Front Hum Neurosci. 2013;7:599. doi: 10.3389/fnhum.2013.00599 24093016 PMC3782703

[38] Schnack HG, Kahn RS. Detecting neuroimaging biomarkers for psychiatric disorders: sample size matters. Front Psych. 2016;7:50. doi: 10.3389/fpsyt.2016.00050 27064972 PMC4814515

[39] Abraham A, Milham MP, Di Martino A, Craddock RC, Samaras D, Thirion B, et al. Deriving reproducible biomarkers from multi-site resting-state data: An Autism-based example. Neuroimage. 2017;147:736–45. doi: 10.1016/j.neuroimage.2016.10.045 27865923

[40] Bonkhoff AK, Schirmer MD, Bretzner M, Hong S, Regenhardt RW, Brudfors M, et al. Outcome after acute ischemic stroke is linked to sex-specific lesion patterns. Nat Commun. 2021 Jun 2;12:3289. doi: 10.1038/s41467-021-23492-3 34078897 PMC8172535

[41] Kiesow H, Spreng RN, Holmes AJ, Chakravarty MM, Marquand AF, Yeo BTT, et al. Deep learning identifies partially overlapping subnetworks in the human social brain. Commun Biol. 2021 Jan 14;4(1):65. doi: 10.1038/s42003-020-01559-z 33446815 PMC7809473

[42] Lanka P, Rangaprakash D, Dretsch MN, Katz JS, Denney TS, Deshpande G. Supervised machine learning for diagnostic classification from large-scale neuroimaging datasets. Brain Imaging Behav. 2019:1–39. doi: 10.1007/s11682-017-9727-6 31691160 PMC7198352

[43] Bzdok D, Floris DL, Marquand AF. Analysing brain networks in population neuroscience: a case for the Bayesian philosophy. Philos Trans R Soc Lond B Biol Sci. 2020;375:20190661. doi: 10.1098/rstb.2019.0661 32089111 PMC7061951

[44] Rosenbaum PR, Rubin DB. The central role of the propensity score in observational studies for causal effects. Biometrika. 1983;70(1):41–55.

[45] Ali MS, Prieto-Alhambra D, Lopes LC, Ramos D, Bispo N, Ichihara MY, et al. Propensity score methods in health technology assessment: principles, extended applications, and recent advances. Front Pharmacol. 2019;10:973. doi: 10.3389/fphar.2019.00973 31619986 PMC6760465

[46] Austin PC. An introduction to propensity score methods for reducing the effects of confounding in observational studies. Multivar Behav Res. 2011;46(3):399–424. doi: 10.1080/00273171.2011.568786 21818162 PMC3144483

[47] Bzdok D, Ioannidis JP. Exploration, inference, and prediction in neuroscience and biomedicine. Trends Neurosci. 2019;42(4):251–62. doi: 10.1016/j.tins.2019.02.001 30808574

[48] Dinga R, Schmaal L, Penninx BW, Veltman DJ, Marquand AF. Controlling for effects of confounding variables on machine learning predictions. bioRxiv. 2020.

[49] Rao A, Monteiro JM, Mourao-Miranda J. Alzheimer’s Disease Initiative. Predictive modelling using neuroimaging data in the presence of confounds. Neuroimage. 2017;150:23–49. doi: 10.1016/j.neuroimage.2017.01.066 28143776 PMC5391990

[50] Wachinger C, Rieckmann A, Pölsterl S, Alzheimer’s Disease Neuroimaging Initiative. Detect and correct bias in multi-site neuroimaging datasets. Med Image Anal. 2020;67:101879. doi: 10.1016/j.media.2020.101879 33152602

[51] Lord C, Rutter M, DiLavore PC. Autism Diagnostic Observation Schedule—Generic. Dissertation Abstracts International Section A: Humanities and Social Sciences 1999;

[52] Lefort-Besnard J, Vogeley K, Schilbach L, Varoquaux G, Thirion B, Dumas G, et al. Patterns of autism symptoms: hidden structure in the ADOS and ADI-R instruments. Transl Psychiatry. 2020 Jul 30;10(1):1–12. doi: 10.1038/s41398-019-0665-5 32732967 PMC7393151

[53] Lord C, Rutter M, Le Couteur A. Autism Diagnostic Interview-Revised: a revised version of a diagnostic interview for caregivers of individuals with possible pervasive developmental disorders. J Autism Dev Disord. 1994;24(5):659–85. doi: 10.1007/BF02172145 7814313

[54] APA. Diagnostic and statistical manual of mental disorders: DSM-5. Washington, DC: Autor; 2013. 10.1590/s2317-17822013000200017 24413388

[55] Dale AM, Fischl B, Sereno MI. Cortical surface-based analysis: I. Segmentation and surface reconstruction. Neuroimage. 1999;9(2):179–94. doi: 10.1006/nimg.1998.0395 9931268

[56] Fischl B. FreeSurfer. Neuroimage. 2012;62(2):774–81. doi: 10.1016/j.neuroimage.2012.01.021 22248573 PMC3685476

[57] Fischl B, Sereno MI, Dale AM. Cortical surface-based analysis: II: inflation, flattening, and a surface-based coordinate system. Neuroimage. 1999;9(2):195–207. doi: 10.1006/nimg.1998.0396 9931269

[58] Behzadi Y, Restom K, Liau J, Liu TT. A component based noise correction method (CompCor) for BOLD and perfusion based fMRI. Neuroimage. 2007;37(1):90–101. doi: 10.1016/j.neuroimage.2007.04.042 17560126 PMC2214855

[59] Andersson JLR, Skare S, Ashburner J. How to correct susceptibility distortions in spin-echo echo-planar images: application to diffusion tensor imaging. Neuroimage. 2003 Oct 1;20(2):870–88. doi: 10.1016/S1053-8119(03)00336-7 14568458

[60] Salimi-Khorshidi G, Douaud G, Beckmann CF, Glasser MF, Griffanti L, Smith SM. Automatic denoising of functional MRI data: combining independent component analysis and hierarchical fusion of classifiers. Neuroimage. 2014;90:449–68. doi: 10.1016/j.neuroimage.2013.11.046 24389422 PMC4019210

[61] Schaefer A, Kong R, Gordon EM, Laumann TO, Zuo X-N, Holmes AJ, et al. Local-global parcellation of the human cerebral cortex from intrinsic functional connectivity MRI. Cereb Cortex. 2018;28(9):3095–114. doi: 10.1093/cercor/bhx179 28981612 PMC6095216

[62] Bishop CM. Pattern recognition and machine learning. Springer; 2006.

[63] Schulz M-A, Yeo BTT, Vogelstein JT, Mourao-Miranada J, Kather JN, Kording K, et al. Different scaling of linear models and deep learning in UKBiobank brain images versus machine-learning datasets. Nat Commun. 2020 Aug 25;11:4238. doi: 10.1038/s41467-020-18037-z 32843633 PMC7447816

[64] Lee BK, Lessler J, Stuart EA. Weight trimming and propensity score weighting. PLoS ONE. 2011;6(3):e18174. doi: 10.1371/journal.pone.0018174 21483818 PMC3069059

[65] Setoguchi S, Schneeweiss S, Brookhart MA, Glynn RJ, Cook EF. Evaluating uses of data mining techniques in propensity score estimation: a simulation study. Pharmacoepidemiol Drug Saf. 2008;17(6):546–55. doi: 10.1002/pds.1555 18311848 PMC2905676

[66] Westreich D, Lessler J, Funk MJ. Propensity score estimation: neural networks, support vector machines, decision trees (CART), and meta-classifiers as alternatives to logistic regression. J Clin Epidemiol. 2010;63(8):826–33. doi: 10.1016/j.jclinepi.2009.11.020 20630332 PMC2907172

[67] Platt JC. Probabilistic Outputs for Support Vector Machines and Comparisons to Regularized Likelihood Methods. In: Advances in Large Margin Classifiers. MIT Press; 1999. p. 61–74.

[68] Stuart EA. Matching methods for causal inference: A review and a look forward. Stat Sci. 2010 Feb 1;25(1):1–21. doi: 10.1214/09-STS313 20871802 PMC2943670

[69] Hedlin H, Caffo B, Mahfoud Z, Bassett SS. Covariate-adjusted nonparametric analysis of magnetic resonance images using Markov chain Monte Carlo. Stat Interface. 2010;3(1):113. doi: 10.4310/sii.2010.v3.n1.a11 22163068 PMC3232683

[70] Linn KA, Gaonkar B, Doshi J, Davatzikos C, Shinohara RT. Addressing confounding in predictive models with an application to neuroimaging. Int J Biostat. 2016;12(1):31–44. doi: 10.1515/ijb-2015-0030 26641972 PMC5154735

[71] McMurry TL, Hu Y, Blackstone EH, Kozower BD. Propensity scores: methods, considerations, and applications in the Journal of Thoracic and Cardiovascular Surgery. J Thorac Cardiovasc Surg. 2015;150(1):14–9. doi: 10.1016/j.jtcvs.2015.03.057 25963441

[72] Kuhn HW. The Hungarian method for the assignment problem. Naval Research Logistics Quarterly. 1955;2(1–2):83–97.

[73] Lunt M. Selecting an Appropriate Caliper Can Be Essential for Achieving Good Balance With Propensity Score Matching. Am J Epidemiol. 2014 Jan 15;179(2):226–35. doi: 10.1093/aje/kwt212 24114655 PMC3873103

[74] Austin PC. Optimal caliper widths for propensity-score matching when estimating differences in means and differences in proportions in observational studies. Pharm Stat. 2011;10(2):150–61. doi: 10.1002/pst.433 20925139 PMC3120982

[75] Wang Y, Cai H, Li C, Jiang Z, Wang L, Song J, et al. Optimal caliper width for propensity score matching of three treatment groups: a Monte Carlo study. PLoS ONE. 2013;8(12):e81045. doi: 10.1371/journal.pone.0081045 24349029 PMC3859481

[76] Alfaro-Almagro F, McCarthy P, Afyouni S, Andersson JL, Bastiani M, Miller KL, et al. Confound modelling in UK Biobank brain imaging. Neuroimage. 2020;224:117002. doi: 10.1016/j.neuroimage.2020.117002 32502668 PMC7610719

[77] Bernardino G, Benkarim O, Sanz-de la Garza M, Prat-Gonzàlez S, Sepulveda-Martinez A, Crispi F, et al. Handling confounding variables in statistical shape analysis—application to cardiac remodelling. Med Image Anal. 2020 Oct 1;65:101792. doi: 10.1016/j.media.2020.101792 32712526

[78] Snoek L, Miletić S, Scholte HS. How to control for confounds in decoding analyses of neuroimaging data. Neuroimage. 2019;184:741–60. doi: 10.1016/j.neuroimage.2018.09.074 30268846

[79] Görgen K, Hebart MN, Allefeld C, Haynes J-D. The same analysis approach: Practical protection against the pitfalls of novel neuroimaging analysis methods. Neuroimage. 2018;180:19–30. doi: 10.1016/j.neuroimage.2017.12.083 29288130 PMC6021230

[80] Johnson WE, Li C, Rabinovic A. Adjusting batch effects in microarray expression data using empirical Bayes methods. Biostatistics. 2007;8(1):118–27. doi: 10.1093/biostatistics/kxj037 16632515

[81] Dukart J, Schroeter ML, Mueller K, Alzheimer’s Disease Neuroimaging Initiative. Age correction in dementia–matching to a healthy brain. PLoS ONE. 2011;6(7):e22193. doi: 10.1371/journal.pone.0022193 21829449 PMC3146486

[82] Fortin J-P, Parker D, Tunç B, Watanabe T, Elliott MA, Ruparel K, et al. Harmonization of multi-site diffusion tensor imaging data. Neuroimage. 2017;161:149–70. doi: 10.1016/j.neuroimage.2017.08.047 28826946 PMC5736019

[83] Larivière S, Rodríguez-Cruces R, Royer J, Caligiuri ME, Gambardella A, Concha L, et al. Network-based atrophy modeling in the common epilepsies: A worldwide ENIGMA study. Sci Adv. 2020 Nov;6(47):eabc6457. doi: 10.1126/sciadv.abc6457 33208365 PMC7673818

[84] Yu M, Linn KA, Cook PA, Phillips ML, McInnis M, Fava M, et al. Statistical harmonization corrects site effects in functional connectivity measurements from multi-site fMRI data. Hum Brain Mapp. 2018 Nov;39(11):4213. doi: 10.1002/hbm.24241 29962049 PMC6179920

[85] Hatton SN, Huynh KH, Bonilha L, Abela E, Alhusaini S, Altmann A, et al. White matter abnormalities across different epilepsy syndromes in adults: an ENIGMA-Epilepsy study. Brain. 2020 Aug 1;143(8):2454–73. doi: 10.1093/brain/awaa200 32814957 PMC7567169

[86] Chyzhyk D, Varoquaux G, Thirion B, Milham M. Controlling a confound in predictive models with a test set minimizing its effect. In IEEE. 2018:1–4.

[87] Yeo BTT, Krienen FM, Sepulcre J, Sabuncu MR, Lashkari D, Hollinshead M, et al. The organization of the human cerebral cortex estimated by intrinsic functional connectivity. J Neurophysiol. 2011;106:1125–65. doi: 10.1152/jn.00338.2011 21653723 PMC3174820

[88] Smith SM, Nichols TE. Threshold-free cluster enhancement: addressing problems of smoothing, threshold dependence and localisation in cluster inference. Neuroimage. 2009;44(1):83–98. doi: 10.1016/j.neuroimage.2008.03.061 18501637

[89] Bzdok D, Meyer-Lindenberg A. Machine learning for precision psychiatry: opportunities and challenges. Biol Psychiatry Cogn Neurosci Neuroimaging. 2018;3(3):223–30. doi: 10.1016/j.bpsc.2017.11.007 29486863

[90] Duncan NW, Northoff G. Overview of potential procedural and participant-related confounds for neuroimaging of the resting state. J Psychiatry Neurosci. 2013;38(2):84. doi: 10.1503/jpn.120059 22964258 PMC3581596

[91] Lord C, Bishop S, Anderson D. Developmental trajectories as autism phenotypes. In Wiley Online. Library. 2015:198–208. doi: 10.1002/ajmg.c.31440 25959391 PMC4898819

[92] Ullman MT, Pullman MY. A compensatory role for declarative memory in neurodevelopmental disorders. Neurosci Biobehav Rev. 2015;51:205–22. doi: 10.1016/j.neubiorev.2015.01.008 25597655 PMC4359651

[93] Khundrakpam BS, Lewis JD, Kostopoulos P, Carbonell F, Evans AC. Cortical thickness abnormalities in autism spectrum disorders through late childhood, adolescence, and adulthood: a large-scale MRI study. Cereb Cortex. 2017;27(3):1721–31. doi: 10.1093/cercor/bhx038 28334080

[94] Nunes AS, Vakorin VA, Kozhemiako N, Peatfield N, Ribary U, Doesburg SM. Atypical age-related changes in cortical thickness in autism spectrum disorder. Sci Rep. 2020;10(1):1–15. doi: 10.1038/s41598-019-56847-4 32632150 PMC7338512

[95] Valk SL, Di Martino A, Milham MP, Bernhardt BC. Multicenter mapping of structural network alterations in autism. Hum Brain Mapp. 2015;36(6):2364–73. doi: 10.1002/hbm.22776 25727858 PMC6129398

[96] Zielinski BA, Prigge MB, Nielsen JA, Froehlich AL, Abildskov TJ, Anderson JS, et al. Longitudinal changes in cortical thickness in autism and typical development. Brain. 2014;137(6):1799–812. doi: 10.1093/brain/awu083 24755274 PMC4032101

[97] Hong SJ, de Wael RV, Bethlehem RAI, Lariviere S, Paquola C, Valk SL, et al. Atypical functional connectome hierarchy in autism. Nature. IDAA Commun. 2019;10(1). doi: 10.1038/s41467-019-08944-1 30833582 PMC6399265

[98] Kozhemiako N, Nunes AS, Vakorin V, Iarocci G, Ribary U, Doesburg SM. Alterations in Local Connectivity and Their Developmental Trajectories in Autism Spectrum Disorder: Does Being Female Matter? Cereb Cortex. 2020;30(9):5166–79. doi: 10.1093/cercor/bhaa109 32368779

[99] Nomi JS, Uddin LQ. Developmental changes in large-scale network connectivity in autism. Neuroimage Clin. 2015;7:732–41. doi: 10.1016/j.nicl.2015.02.024 25844325 PMC4375789

[100] Vigneshwaran S, Mahanand BS, Suresh S, Sundararajan N. Using regional homogeneity from functional MRI for diagnosis of ASD among males. In IEEE. 2015:1–8.

[101] Alaerts K, Swinnen SP, Wenderoth N. Sex differences in autism: a resting-state fMRI investigation of functional brain connectivity in males and females. Soc Cogn Affect Neurosci. 2016;11(6):1002–16. doi: 10.1093/scan/nsw027 26989195 PMC4884321

[102] Lawrence KE, Hernandez LM, Bookheimer SY, Dapretto M. Atypical longitudinal development of functional connectivity in adolescents with autism spectrum disorder. Autism Res. 2019;12(1):53–65. doi: 10.1002/aur.1971 30375176 PMC6325013

[103] Retico A, Giuliano A, Tancredi R, Cosenza A, Apicella F, Narzisi A, et al. The effect of gender on the neuroanatomy of children with autism spectrum disorders: a support vector machine case-control study. Mol Autism. 2016;7(1):1–20. doi: 10.1186/s13229-015-0067-3 26788282 PMC4717545

[104] Blumberg SJ, Bramlett MD, Kogan MD, Schieve LA, Jones JR, Lu MC. Changes in prevalence of parent-reported autism spectrum disorder in school-aged US children: 2007 to 2011–2012. US Department of Health and Human Services, Centers for Disease Control and …; 2013. 24988818

[105] Halladay AK, Bishop S, Constantino JN, Daniels AM, Koenig K, Palmer K, et al. Sex and gender differences in autism spectrum disorder: summarizing evidence gaps and identifying emerging areas of priority. Mol Autism. 2015 Jun 13;6(1):36. doi: 10.1186/s13229-015-0019-y 26075049 PMC4465158

[106] Werling DM, Geschwind DH. Sex differences in autism spectrum disorders. Curr Opin Neurol. 2013 Apr;26(2):146–53. doi: 10.1097/WCO.0b013e32835ee548 23406909 PMC4164392

[107] Ramtekkar UP, Reiersen AM, Todorov AA, Todd RD. Sex and age differences in attention-deficit/hyperactivity disorder symptoms and diagnoses: implications for DSM-V and ICD-11. J Am Acad Child Adolesc Psychiatry. 2010;49(3):217–228. e3. 20410711 PMC3101894

[108] McLean CP, Asnaani A, Litz BT, Hofmann SG. Gender differences in anxiety disorders: prevalence, course of illness, comorbidity and burden of illness. J Psychiatr Res. 2011;45(8):1027–35. doi: 10.1016/j.jpsychires.2011.03.006 21439576 PMC3135672

[109] Colby JB, Rudie JD, Brown JA, Douglas PK, Cohen MS, Shehzad Z. Insights into multimodal imaging classification of ADHD. Front Syst Neurosci. 2012;6:59. doi: 10.3389/fnsys.2012.00059 22912605 PMC3419970

[110] Spera G, Retico A, Bosco P, Ferrari E, Palumbo L, Oliva P, et al. Evaluation of altered functional connections in male children with autism spectrum disorders on multiple-site data optimized with machine learning. Front Psych. 2019;10:620. doi: 10.3389/fpsyt.2019.00620 31616322 PMC6763745

[111] Gogtay N, Giedd JN, Lusk L, Hayashi KM, Greenstein D, Vaituzis AC, et al. Dynamic mapping of human cortical development during childhood through early adulthood. Proc Natl Acad Sci U S A. 2004 May 25;101(21):8174–9. doi: 10.1073/pnas.0402680101 15148381 PMC419576

[112] Wierenga LM, van den Heuvel MP, van Dijk S, Rijks Y, de Reus MA, Durston S. The development of brain network architecture. Hum Brain Mapp. 2016 Feb;37(2):717–29. doi: 10.1002/hbm.23062 26595445 PMC6867575

[113] Mueller S, Wang D, Fox MD, Yeo BTT, Sepulcre J, Sabuncu MR, et al. Individual Variability in Functional Connectivity Architecture of the Human Brain. Neuron. 2013;77:586–95. doi: 10.1016/j.neuron.2012.12.028 23395382 PMC3746075

[114] Glasser MF, Van Essen DC. Mapping human cortical areas in vivo based on myelin content as revealed by T1- and T2-weighted MRI. J Neurosci 2011 Aug 10;31(32):11597–616. doi: 10.1523/JNEUROSCI.2180-11.2011 21832190 PMC3167149

[115] Timmler S, Simons M. Grey matter myelination. Glia. 2019;67(11):2063–70. doi: 10.1002/glia.23614 30860619

[116] Turner R. Myelin and Modeling: Bootstrapping Cortical Microcircuits. Front Neural Circuits. 2019;13:34. doi: 10.3389/fncir.2019.00034 31133821 PMC6517540

[117] Karahan E, Tait L, Si R, Özkan A, Szul M, Zhang J. Individual variability in the human connectome maintains selective cross-modal consistency and shares microstructural signatures [Internet]. bioRxiv; 2021 [cited 2022 Apr 13]. p. 2021.04.01.438129. Available from: https://www.biorxiv.org/content/10.1101/2021.04.01.438129v1.

[118] Assaf M, Jagannathan K, Calhoun VD, Miller L, Stevens MC, Sahl R, et al. Abnormal functional connectivity of default mode sub-networks in autism spectrum disorder patients. Neuroimage. 2010;53(1):247–56. doi: 10.1016/j.neuroimage.2010.05.067 20621638 PMC3058935

[119] Di Martino A, Ross K, Uddin LQ, Sklar AB, Castellanos FX, Milham MP. Functional brain correlates of social and nonsocial processes in autism spectrum disorders: an activation likelihood estimation meta-analysis. Biol Psychiatry. 2009;65(1):63–74. doi: 10.1016/j.biopsych.2008.09.022 18996505 PMC2993772

[120] Farrant K, Uddin LQ. Atypical developmental of dorsal and ventral attention networks in autism. Dev Sci. 2016;19(4):550–63. doi: 10.1111/desc.12359 26613549

[121] Fitzgerald J, Johnson K, Kehoe E, Bokde AL, Garavan H, Gallagher L, et al. Disrupted functional connectivity in dorsal and ventral attention networks during attention orienting in autism spectrum disorders. Autism Res. 2015;8(2):136–52. doi: 10.1002/aur.1430 25428212

[122] Just MA, Keller TA, Malave VL, Kana RK, Varma S. Autism as a neural systems disorder: a theory of frontal-posterior underconnectivity. Neurosci Biobehav Rev. 2012;36(4):1292–313. doi: 10.1016/j.neubiorev.2012.02.007 22353426 PMC3341852

[123] Kennedy DP, Courchesne E. The intrinsic functional organization of the brain is altered in autism. Neuroimage. 2008;39(4):1877–85. doi: 10.1016/j.neuroimage.2007.10.052 18083565

[124] Müller R-A, Shih P, Keehn B, Deyoe JR, Leyden KM, Shukla DK. Underconnected, but how? A survey of functional connectivity MRI studies in autism spectrum disorders. Cereb Cortex. 2011;21(10):2233–43. doi: 10.1093/cercor/bhq296 21378114 PMC3169656

[125] Kernbach JM, Satterthwaite TD, Bassett DS, Smallwood J, Margulies D, Krall S, et al. Shared endo-phenotypes of default mode dsfunction in attention deficit/hyperactivity disorder and autism spectrum disorder. Transl Psychiatry. 2018 Jul 17;8(1):133. doi: 10.1038/s41398-018-0179-6 30018328 PMC6050263

[126] Padmanabhan A, Lynch CJ, Schaer M, Menon V. The default mode network in autism. Biol Psychiatry Cogn Neurosci Neuroimaging. 2017;2(6):476–86. doi: 10.1016/j.bpsc.2017.04.004 29034353 PMC5635856

[127] Benkarim O, Paquola C, Park B, Hong S-J, Royer J, Vos de Wael R, et al. Connectivity alterations in autism reflect functional idiosyncrasy. Commun Biol. 2021 Sep 15;4(1):1–15. doi: 10.1038/s42003-020-01566-0 34526654 PMC8443598

[128] Dickie EW, Ameis SH, Shahab S, Calarco N, Smith DE, Miranda D, et al. Personalized intrinsic network topography mapping and functional connectivity deficits in autism spectrum disorder. Biol Psychiatry. 2018;84(4):278–86. doi: 10.1016/j.biopsych.2018.02.1174 29703592 PMC6076333

[129] Nunes AS, Peatfield N, Vakorin V, Doesburg SM. Idiosyncratic organization of cortical networks in autism spectrum disorder. Neuroimage. 2019;190:182–90. doi: 10.1016/j.neuroimage.2018.01.022 29355768

[130] Bia M, Mattei A. A Stata package for the estimation of the dose-response function through adjustment for the generalized propensity score. Stata J. 2008;8(3):354–73.

[131] Hirano K, Imbens GW. The propensity score with continuous treatments. Applied Bayesian modeling and causal inference from incomplete-data perspectives. 2004;226164:73–84.

[132] Austin PC. Assessing the performance of the generalized propensity score for estimating the effect of quantitative or continuous exposures on binary outcomes. Stat Med. 2018;37(11):1874–94. doi: 10.1002/sim.7615 29508424 PMC5969262

[133] Moradi E, Khundrakpam B, Lewis JD, Evans AC, Tohka J. Predicting symptom severity in autism spectrum disorder based on cortical thickness measures in agglomerative data. Neuroimage. 2017;144:128–41. doi: 10.1016/j.neuroimage.2016.09.049 27664827

[134] Quiñonero-Candela J, Sugiyama M, Schwaighofer A, Lawrence ND, editors. Dataset Shift in Machine Learning. Cambridge, MA, USA: MIT Press; 2008. 248 p. (Jordan MI, Dietterich TG, editors. Neural Information Processing series).

[135] Castro DC, Walker I, Glocker B. Causality matters in medical imaging. Nat Commun 2020 Jul 22;11(1):3673. doi: 10.1038/s41467-020-17478-w 32699250 PMC7376027

[136] Moreno-Torres JG, Raeder T, Alaiz-Rodríguez R, Chawla NV, Herrera F. A unifying view on dataset shift in classification. Pattern Recogn 2012 Jan 1;45(1):521–30.

[137] Subbaswamy A, Saria S. From development to deployment: dataset shift, causality, and shift-stable models in health AI. Biostatistics 2020 Apr 1;21(2):345–52. doi: 10.1093/biostatistics/kxz041 31742354

[138] Neto EC. Causality-aware counterfactual confounding adjustment as an alternative to linear residualization in anticausal prediction tasks based on linear learners. arXiv:201104605 [cs, stat] [Internet]. 2020 Nov 9 [cited 2022 Apr 13]; Available from: http://arxiv.org/abs/2011.04605.

[139] Pearl J. Theoretical Impediments to Machine Learning With Seven Sparks from the Causal Revolution. In: Proceedings of the Eleventh ACM International Conference on Web Search and Data Mining [Internet] New York, NY, USA: Association for Computing Machinery; 2018 [cited 2022 Apr 13]. p. 3. (WSDM ‘18). Available from: 10.1145/3159652.3176182.

[140] Pearl J. Causality: Models, reasoning, and inference. New York, NY, US: Cambridge University Press; 2000. xvi, 384 p. (Causality: Models, reasoning, and inference).

[141] Wang Y, Blei DM. The Blessings of Multiple Causes. J Am Stat Assoc. 2019 Oct 2;114(528):1574–96.

[142] Stürmer T, Rothman KJ, Avorn J, Glynn RJ. Treatment Effects in the Presence of Unmeasured Confounding: Dealing With Observations in the Tails of the Propensity Score Distribution—A Simulation Study. Am J Epidemiol. 2010 Oct 1;172(7):843–54. doi: 10.1093/aje/kwq198 20716704 PMC3025652

[143] Tanaka SC, Yamashita A, Yahata N, Itahashi T, Lisi G, Yamada T, et al. A multi-site, multi disorder resting-state magnetic resonance image database. Sci Data. 2021 Aug 30;8(1):227. doi: 10.1038/s41597-021-01004-8 34462444 PMC8405782

