# Supplementary material for: Population heterogeneity in clinical cohorts affects the predictive accuracy of brain imaging
Source: PLoS Biol. 2022 Apr 29;20(4):e3001627. doi: 10.1371/journal.pbio.3001627 (PMC9094526; doi:10.1371/journal.pbio.3001627)
Supplement: S1 Table — CBIC, CitiGroup Corcell Brain Imaging Center; FA, flip angle; NYU, New York University Langone Medical Center; PITT, University of Pittsburgh, School of Medicine; RU, Rutgers University Brain Imaging Center; SI, Staten Island; TCD, Trinity Centre for Health Sciences, Trinity College Dublin; TE, echo time; TI, inversion time; TR, repetition time; USM, University of Utah, School of Medicine. (DOCX) [file pbio.3001627.s001.docx]

|  | **Site** | **Scanner** | **Modality** | **Sequence** | **TR**  **(mm)** | **TE**  **(mm)** | **TI**  **(mm)** | **FA** | **Voxel**  **(mm^3^)** |
| --- | --- | --- | --- | --- | --- | --- | --- | --- | --- |
| ABIDE | NYU | Siemens Allegra | T1w | 3D-TurboFLASH | 2530 | 3.25 | 1100 | 7° | 1.0×1.0×1.3 |
|  |  |  | fMRI | 2D-EPI | 2000 | 15.00 |  | 90° | 3.0×3.0×4.0 |
|  | PITT | Siemens Allegra | T1w | 3D-MPRAGE | 2100 | 3.93 | 1000 | 7° | 1.1×1.1×1.1 |
|  |  |  | fMRI | 2D-EPI | 1500 | 35.00 |  | 70° | 3.1×3.1×4.0 |
|  | TCD | Philips Achieva | T1w | 3D-MPRAGE | 3000 | 3.90 | 1150 | 8° | 0.9×0.9×0.9 |
|  |  |  | fMRI | 2D-EPI | 2000 | 27.00 |  | 90° | 3.0×3.0×3.2 |
|  | USM | Siemens TrioTim | T1w | 3D-MPRAGE | 2300 | 2.91 | 900 | 9° | 1.0×1.0×1.2 |
|  |  |  | fMRI | 2D-EPI | 2000 | 28.00 |  | 90° | 3.4×3.4×3.0 |
| HBN | CIBIC | Siemens Prisma | T1w | 3D-MPRAGE | 2500 | 3.15 | 1060 | 8° | 0.8×0.8×0.8 |
|  |  |  | fMRI | 2D-EPI | 800 | 30.00 |  | 31° | 2.4×2.4×2.0 |
|  | RU | Siemens TrioTim | T1w | 3D-MPRAGE | 2500 | 3.15 | 1060 | 8° | 0.8×0.8×0.8 |
|  |  |  | fMRI | 2D-EPI | 800 | 30.00 |  | 31° | 2.4×2.4×2.0 |
|  | SI | Siemens Avanto | T1w | 3D-MPRAGE | 2730 | 1.64 | 1000 | 7° | 1.0×1.0×1.0 |
|  |  |  | fMRI | 2D-EPI | 1450 | 40.00 |  | 55° | 2.5×2.5×2.5 |
