# Supplementary material for: Population heterogeneity in clinical cohorts affects the predictive accuracy of brain imaging
Source: PLoS Biol. 2022 Apr 29;20(4):e3001627. doi: 10.1371/journal.pbio.3001627 (PMC9094526; doi:10.1371/journal.pbio.3001627)
Supplement: S2 Table — Number of participants (N), males/females (M/F), and mean age and standard deviation for each site and group. Note that matching was performed separately for each of the different psychiatric conditions (i.e., COND) in HBN. CBIC, CitiGroup Corcell Brain Imaging Center; HBN, Healthy Brain Network; NYU, New York University Langone Medical Center; PITT, University of Pittsburgh, School of Medicine; RU, Rutgers University Brain Imaging Center; SI, Staten Island; TCD, Trinity Centre for Health Sciences, Trinity College Dublin; USM, University of Utah, School of Medicine. (DOCX) [file pbio.3001627.s002.docx]

| **Site** | | **N** | | **Sex (M/F)** | | **Age** | |
| --- | --- | --- | --- | --- | --- | --- | --- |
|  |  | **TD** | **COND** | **TD** | **COND** | **TD** | **COND** |
| **ABIDE** | **NYU** | 68 | 56 | 67/1 | 52/4 | 15.7±6.9 | 14.5±7.9 |
|  | **PITT** | 22 | 20 | 22/- | 20/- | 19.7±7.0 | 20.8±7.3 |
|  | **TCD** | 19 | 18 | 19/- | 18/- | 15.8±3.2 | 14.5±3.3 |
|  | **USM** | 36 | 51 | 36/- | 52/- | 22.7±7.0 | 23.4±7.6 |
|  | **All** | 145 | 145 | 144/1 | 141/4 | 18.1±7.2 | 18.5±8.3 |
| **HBN (ASD)** | **CBIC** | 18 | 17 | 11/12 | 13/4 | 11.5±3.2 | 11.0±2.6 |
|  | **RU** | 30 | 30 | 19/14 | 17/13 | 11.6±3.4 | 12.3±4.1 |
|  | **SI** | 15 | 16 | 10/5 | 11/5 | 12.9±4.2 | 12.6±3.4 |
|  | **All** | 63 | 63 | 40/23 | 41/22 | 11.9±3.6 | 12.0±3.6 |
| **HBN (ADHD)** | **CBIC** | 23 | 29 | 11/12 | 11/18 | 11.7±3.3 | 11.7±3.1 |
|  | **RU** | 33 | 27 | 19/14 | 13/14 | 11.6±3.5 | 12.1±3.4 |
|  | **SI** | 34 | 34 | 20/14 | 20/14 | 12.5±3.8 | 12.9±4.2 |
|  | **All** | 90 | 90 | 50/40 | 44/46 | 12.0±3.6 | 12.3±3.6 |
| **HBN (ANX)** | **CBIC** | 23 | 26 | 11/12 | 14/12 | 11.7±3.3 | 10.9±3.6 |
|  | **RU** | 33 | 34 | 19/14 | 18/16 | 11.6±3.5 | 11.1±3.7 |
|  | **SI** | 38 | 34 | 20/18 | 17/17 | 12.5±3.6 | 12.0±3.5 |
|  | **All** | 94 | 94 | 50/44 | 49/45 | 12.0±3.5 | 11.4±3.6 |
