# Supplementary figures and images for: Population heterogeneity in clinical cohorts affects the predictive accuracy of brain imaging
Source: PLoS Biol. 2022 Apr 29;20(4):e3001627. doi: 10.1371/journal.pbio.3001627 (PMC9094526; doi:10.1371/journal.pbio.3001627)

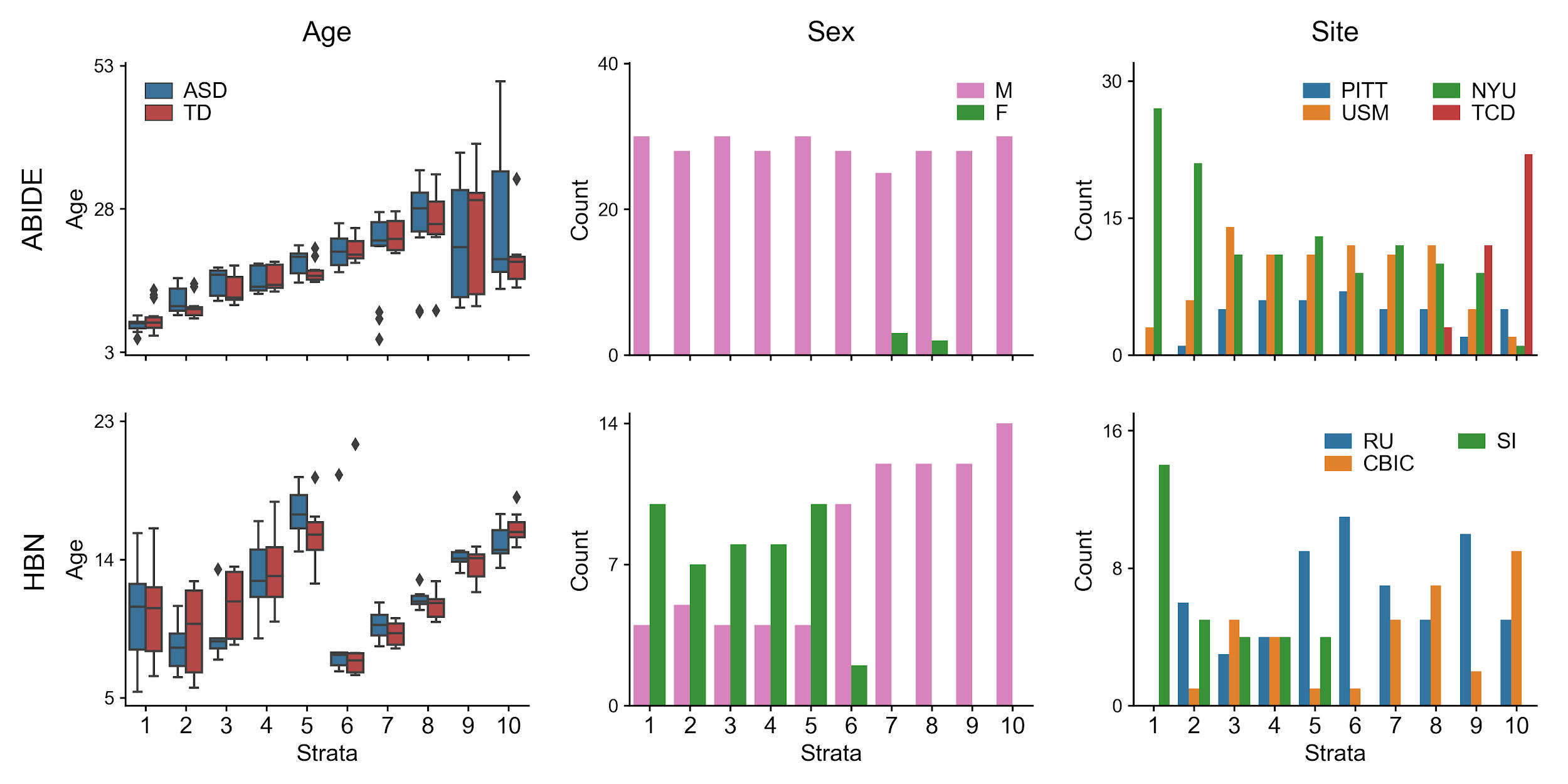

Supplement: S2 Fig — From left to right, distribution of age, sex, and site across strata in ABIDE (top) and HBN (bottom) datasets. The fact that, in HBN, we have females at only one extreme of the propensity score spectrum (i.e., in the strata with the lowest propensity scores) corroborates the validity of our approach to characterize diversity. Data underlying this figure can be found in S1 Data. ABIDE, Autism Brain Imaging Data Exchange; HBN, Healthy Brain Network. (TIF) [file pbio.3001627.s004.tif]

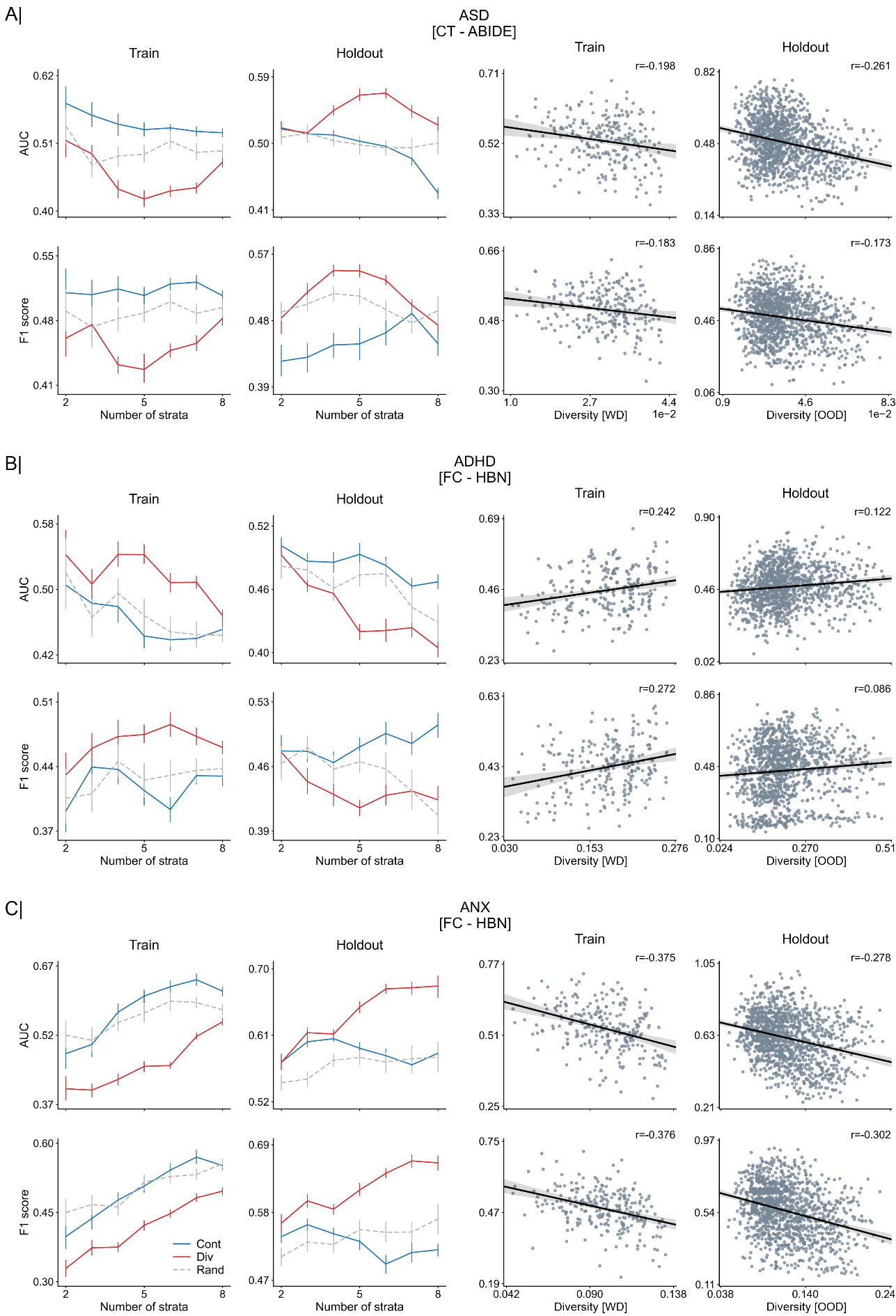

Supplement: S5 Fig — Left: comparison of model performance based on contiguous (Cont) and diverse (Div) training sets in the classification of ASD (top) using CT in ABIDE and ADHD (middle) and ANX (bottom) based on functional connectivity in HBN. Performance is assessed using different sizes of training data (from 2 to 8 combined strata). For each disorder, the first column reports the performance using a 10-fold CV strategy based solely on the training data, whereas the second column displays the performance in the holdout set, which contains the remaining participants (from untouched strata). An additional model (i.e., Rand) is used as a baseline, where training participants are randomly chosen regardless of their diversity (propensity scores). Right: prediction performance based on all possible combinations of 5 out of 10 strata for training (and the remaining 5 for holdout). The first column reports the predictive model performance using a 10-fold CV strategy based solely on the training set, where diversity is computed as the average of all pairwise absolute differences in propensity scores (i.e., WD). The second column displays the performance for each single stratum in the holdout dataset, and diversity denotes the mean absolute difference in propensity scores between the participants of the training set and those in the held-out participants (i.e., OOD). The strength of the association between performance and diversity is reported with Pearson correlation coefficient (r). Data underlying this figure can be found in S1 Data. ABIDE, Autism Brain Imaging Data Exchange; ADHD, attention-deficit/hyperactivity disorder; ASD, autism spectrum disorder; CT, cortical thickness; CV, cross-validation; HBN, Healthy Brain Network; OOD, out of distribution; WD, within distribution. (TIF) [file pbio.3001627.s007.tif]

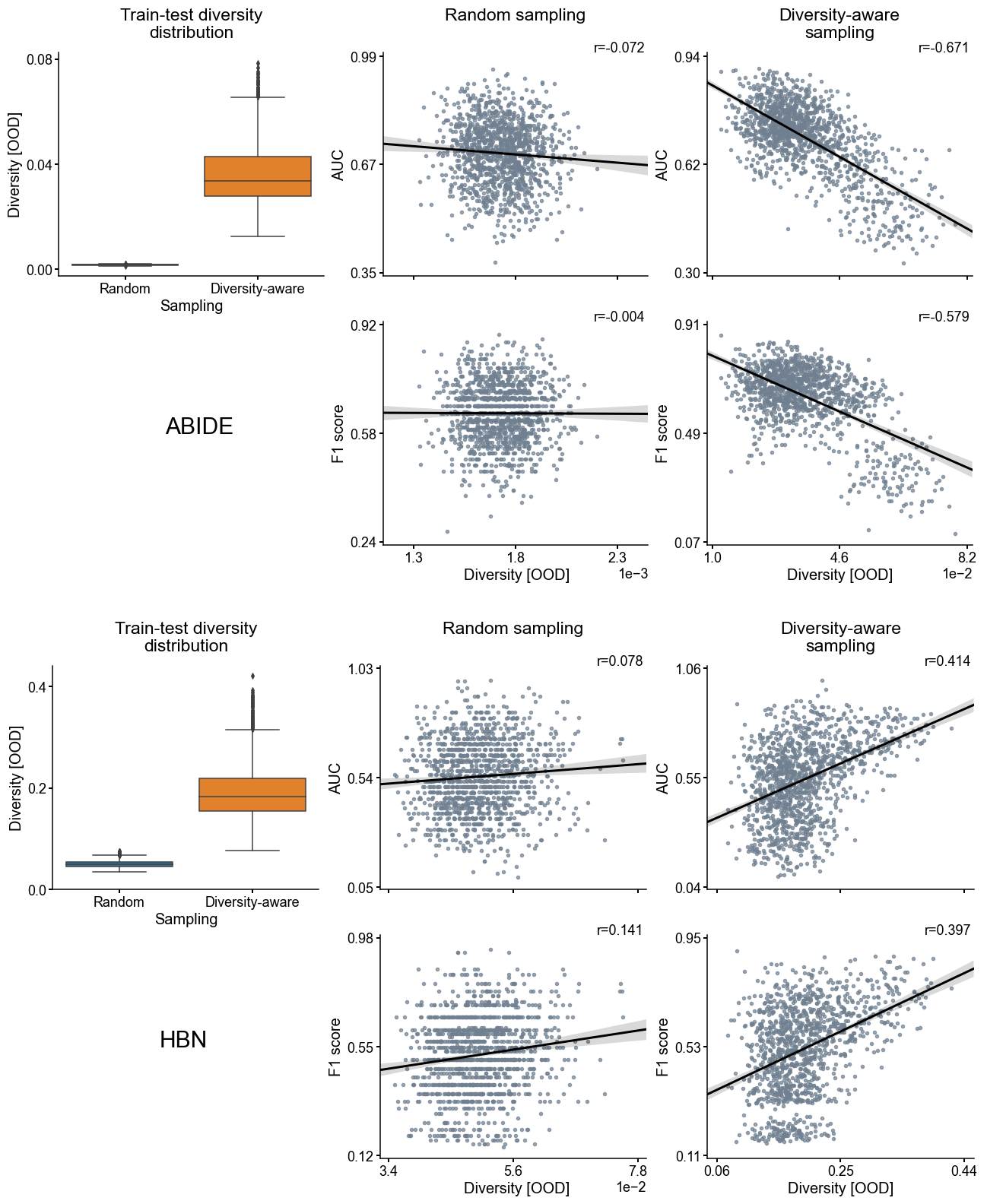

Supplement: S8 Fig — We repeated the out-of-distribution analysis in Fig 3 using the same number of draws but ignoring the strata (i.e., Random sampling) and compared the results to the original sampling strategy based on stratification (i.e., Diversity-aware sampling). Results are shown for ABIDE (top) and HBN (bottom) for both AUC and F1 score performance metrics. Left: distributions of diversity between the train and test subsets for each draw (i.e., data split) based on random (blue) and our diversity-aware sampling (orange). Relationship of diversity with OOD prediction performance when using random (middle) and diversity-aware sampling (right). Ignoring the strata (i.e., random sampling) produces train–test splits with practically no difference in their covariates, which, in turn, does not allow us to analyze the impact of diversity in performance. On the other hand, with stratification, we were able to perform a diversity-aware sampling that produces train–test splits with different covariates. Data underlying this figure can be found in S1 Data. ABIDE, Autism Brain Imaging Data Exchange; AUC, area under the curve; HBN, Healthy Brain Network; OOD, out of distribution. (TIF) [file pbio.3001627.s010.tif]
